# Comparison of Sales From Vending Machines With 4 Different Food and Beverage Messages: A Randomized Trial

**DOI:** 10.1001/jamanetworkopen.2024.9438

**Published:** 2024-05-08

**Authors:** Laura A. Gibson, Alisa J. Stephens-Shields, Sophia V. Hua, Jennifer A. Orr, Hannah G. Lawman, Sara N. Bleich, Kevin G. Volpp, Amy Bleakley, Anne N. Thorndike, Christina A. Roberto

**Affiliations:** 1 Department of Medical Ethics and Health Policy, University of Pennsylvania Perelman School of Medicine, Philadelphia; 2 Center for Health Incentives and Behavioral Economics at the University of Pennsylvania’s Leonard Davis Institute of Health Economics, Philadelphia; 3 Department of Biostatistics, Epidemiology and Informatics, University of Pennsylvania Perelman School of Medicine, Philadelphia; 4 Division of Chronic Disease and Injury Prevention, Philadelphia Department of Public Health, Philadelphia, Pennsylvania; 5 Now with Novo Nordisk Inc, Plainsboro Township, New Jersey; 6 Department of Health Policy & Management, Harvard T.H. Chan School of Public Health, Boston, Massachusetts; 7 Department of Health Care Management, University of Pennsylvania Wharton School, Philadelphia; 8 Department of Communication, University of Delaware, Newark; 9 Division of General Internal Medicine, Massachusetts General Hospital, Boston; 10 Harvard Medical School, Boston, Massachusetts

## Abstract

**Importance:**

Point-of-sale food messaging can encourage healthier purchases, but no studies have directly compared multiple interventions in the field.

**Objective:**

To examine which of 4 food and beverage messages would increase healthier vending machine purchases.

**Design, Setting, and Participants:**

This randomized trial assessed 13 months (February 1, 2019, to February 29, 2020) of vending sales data from 267 machines and 1065 customer purchase assessments from vending machines on government property in Philadelphia, Pennsylvania. Data analysis was performed from March 5, 2020, to November 8, 2022.

**Interventions:**

Study interventions were 4 food and beverage messaging systems: (1) beverage tax posters encouraging healthy choices because of the Philadelphia tax on sweetened drinks; (2) green labels for healthy products; (3) traffic light labels: green (healthy), yellow (moderately healthy), or red (unhealthy); or (4) physical activity equivalent labels (minutes of activity to metabolize product calories).

**Main Outcomes and Measures:**

Sales data were analyzed separately for beverages and snacks. The main outcomes analyzed at the transaction level were calories sold and the health status (using traffic light criteria) of each item sold. Additional outcomes were analyzed at the monthly machine level: total units sold, calories sold, and units of each health status sold. The customer purchase assessment outcome was calories purchased per vending trip.

**Results:**

Monthly sales data came from 150 beverage and 117 snack vending machines, whereas 1065 customers (558 [52%] male) contributed purchase assessment data. Traffic light labels led to a 30% decrease in the mean monthly number of unhealthy beverages sold (mean ratio [MR], 0.70; 95% CI, 0.55-0.88) compared with beverage tax posters. Physical activity labels led to a 34% (MR, 0.66; 95% CI, 0.51-0.87) reduction in the number of unhealthy beverages sold at the machine level and 35% (MR, 0.65; 95% CI, 0.50-0.86) reduction in mean calories sold. Traffic light labels also led to a 30-calorie reduction (b = −30.46; 95% CI, −49.36 to −11.56) per customer trip in the customer purchase analyses compared to physical activity labels. There were very few significant differences for snack machines.

**Conclusions and Relevance:**

In this 13-month randomized trial of 267 vending machines, the traffic light and physical activity labels encouraged healthier beverage purchases, but no change in snack sales, compared with a beverage tax poster. Corporations and governments should consider such labeling approaches to promote healthier beverage choices.

**Trial Registration:**

ClinicalTrials.gov Identifier: NCT06260176

## Key Points

**Question:**

What was the relative effectiveness of 4 food and beverage messages on vending machine sales and calories purchased?

**Findings:**

In this randomized trial of 267 vending machines, machines labeled with physical activity calorie equivalents or traffic lights had significantly lower sales of unhealthy beverages (34% and 30%, respectively) compared with machines with beverage tax posters. Traffic lights compared with physical activity labels significantly decreased total calories purchased among 1065 customers (147 vs 178 kcal).

**Meaning:**

Traffic light and physical activity calorie equivalent labels encouraged healthier beverage, but not snack, purchases compared with a poster about a beverage tax.

## Teaser

This randomized trial uses vending machine data to examine the relative effects of food and beverage messages on vending machine sales and calories purchased.

## Introduction

Healthy diets are associated with decreased risk of cardiovascular disease, obesity, type 2 diabetes, dental caries, and certain cancers.^1^ For this reason, more than 20 countries^2,3,4,5^ have implemented front-of-package food labeling systems to help consumers identify healthier foods, and the US Food and Drug Administration (FDA) is currently conducting research to inform a front-of-package food labeling system in the US. Front-of-package food labels tend to be either numeric based (eg, display percent daily value information) or interpretive, meaning they display more intuitive, nonnumeric information to help consumers judge the product’s healthfulness (eg, warning labels and traffic light labels). Numerous laboratory and online experiments find that interpretive front-of-package labels encourage people to buy healthier products,^6^ but only a handful of natural field experiments have examined label effects using objective sales data, and no field experiments have compared different types of interpretive label designs.^5^

The existing literature provides evidence of the effectiveness of traffic light labels in cafeteria and community supermarket settings,^7,8^ physical activity calorie equivalent labels in corner stores,^9,10^ a Guiding Stars symbol that gives foods a 0 to 3 score in supermarkets,^11^ and nutrient warnings implemented nationwide in Chile.^12,13^ Additionally, a few studies have revealed mixed results or small effects for point-of-sale nutrition labels in vending machines,^14^ but these studies only tested a small number of machines (3-18) for a short period (<6 months).^15,16,17,18,19^ One study with more machines, but still for a short period, showed no effect of traffic light labels after increasing the availability of healthy products.^20^ The largest previous food labeling study with vending machines randomized 55 snack machines to 1 of 12 treatments (3 labeling changes [none, low-fat label, or low-fat label plus signage] by 4 pricing changes [equal, 10%, 25%, or 50% reductions] for low-fat snacks) for 4 weeks in a Latin square sequence for 12 months. When aggregated across price conditions, the low-fat label plus signage led to an increase in the sale of low-fat snacks from 14% to 15% compared with no label.^21^

There is a lack of field studies directly comparing which label designs are most effective at influencing behavior. Therefore, this study aimed to test the relative effectiveness of 4 food and beverage messages on vending machine sales and individual customer purchases. To accomplish this aim, we partnered with the Philadelphia Department of Public Health to randomize 267 vending machines located on Philadelphia government property into 1 of 4 food and beverage messaging conditions: (1) sweetened beverage tax messaging (poster only); (2) green labels (healthy; placed only on healthier items); (3) traffic light labels: green (healthy), yellow (moderately healthy), or red (unhealthy) (placed on all items); or (4) physical activity calorie equivalent labels (placed on all items). All conditions displayed a poster explaining how to interpret the labels. To determine whether the messages led to differences by condition in the average healthfulness of the products sold, we analyzed the sales data in 2 ways. First, we conducted transaction-level analyses to determine whether the messaging impacted what people purchased during a vending machine transaction. Second, we conducted machine-level analyses to understand whether the messaging led customers to abstain from purchasing altogether. To complement the sales data, we also collected individual-level customer purchases by recruiting people after their purchases and recording the items they bought.

Because people cite cost, convenience, and taste as the main drivers of food purchases^22^ and studies have found that making taxes salient through point-of-sale signage increases their behavioral impact,^23,24^ we hypothesized that posters reminding customers of the beverage tax would lead to the greatest improvements in dietary choices. We also hypothesized that traffic light labels would exert greater behavioral influence than the green labels only on healthier items because people tend to give greater weight to negative entities (ie, events and messages) compared with positive ones.^25^ Finally, we hypothesized that the physical activity labels would also perform better than the green labels given a recent meta-analysis of 15 studies showing that such labels reduced calories selected and consumed compared with no label.^26^

## Methods

### Study Setting

This randomized trial was conducted in Philadelphia, Pennsylvania. Of the 10 largest US cities, Philadelphia has the largest proportion of residents living in poverty and has the worst chronic disease profile, making it an important place to improve dietary choices.^27,28,29^ This work was performed in partnership with the Philadelphia Department of Public Health. In 2013, the Philadelphia Department of Public Health implemented healthy vending standards, requiring that 65% of the items offered in vending machines on city property be healthy and labeled as such.^30^ Compliance with those standards was completed by 2018, before the start of this study.^31^ Philadelphia also implemented a 1.5 cent-per-ounce sweetened beverage tax in 2017, which remained stable during our study period. This study was deemed exempt from review by the University of Pennsylvania Institutional Review Board because it used anonymous survey and observational procedures. The trial protocol can be found in Supplement 1. The study followed the Consolidated Standards of Reporting Trials (CONSORT) reporting guideline.

### Vending Machine Sample

Vending machines located on city property were randomized to 1 of the 4 food and beverage message conditions in parallel stratified by 1 of 5 location types (correctional facilities, courts or offices, large offices, police or fire stations, and recreation centers, libraries, or other) and baseline machine sales at that location (high or low). We randomized at the vending machine floor level to prevent spillover effects among machines on the same floor. There were 150 beverage machines in 108 randomization locations and 117 snack machines in 100 randomization locations (Figure), accessible to 26 000 employees and the public.

**Figure.**
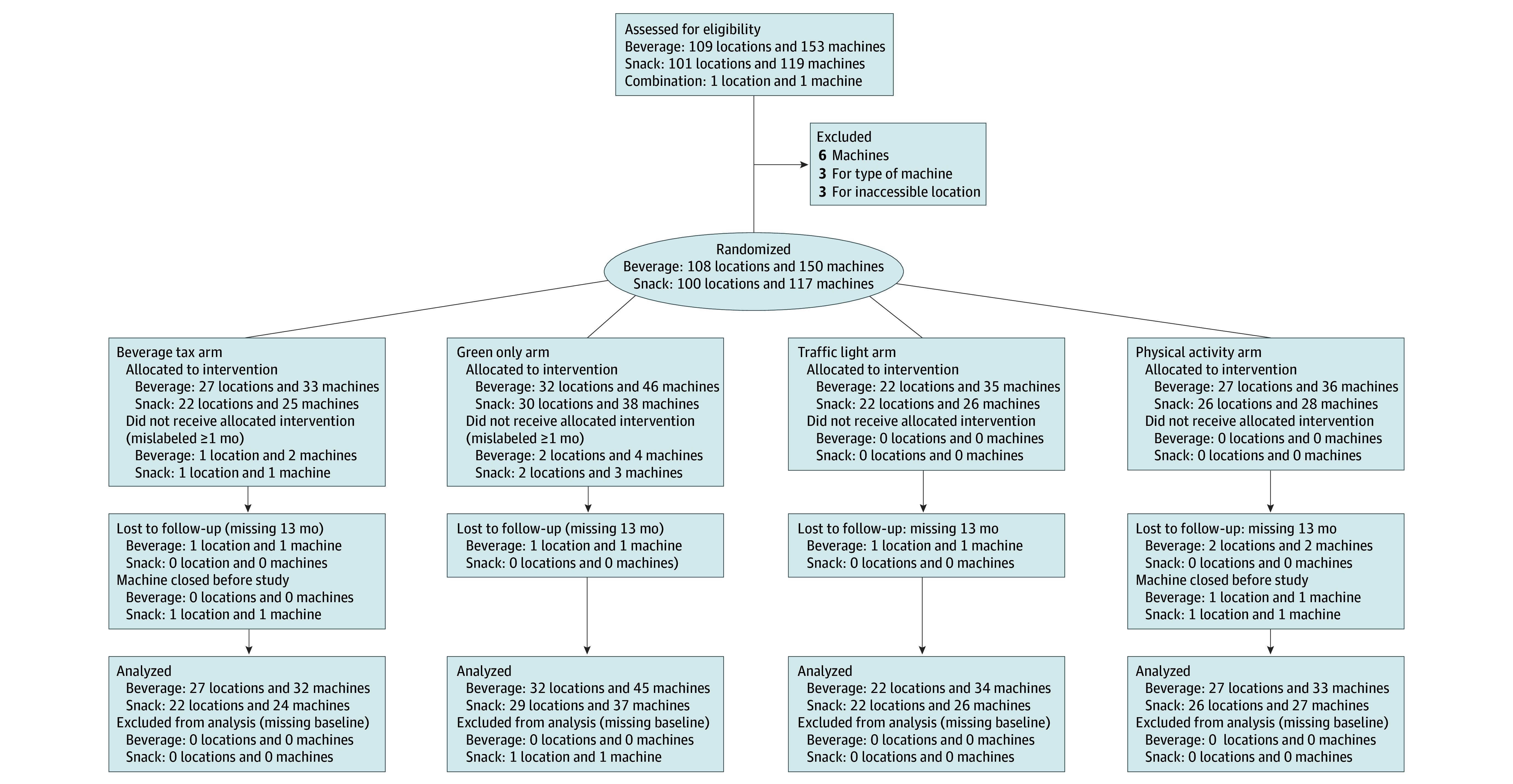
Study Flow

### Intervention

The 4 food and beverage messaging interventions were as follows: (1) beverage tax: no product-specific labels, just beverage machine posters stating, “Philly bev tax is here. Starting January 1, 2017, a 1.5-cents-per-ounce tax will be applied to sweetened beverages.” or snack machine posters stating, “Are you drinking fewer sweetened drinks because of the cost? Keep up the healthy choices by also choosing healthy snacks.”; (2) green only: a green label only on items meeting the “healthy” threshold; (3) traffic light: a green (healthy), yellow (moderately healthy), or red (unhealthy) label on every item; or (4) physical activity: a physical activity calorie equivalent label on every item that indicated the number of minutes one would have to walk to metabolize the calories in the item. eAppendix 1 in Supplement 2 shows all posters and labels and explains the nutrition traffic light assignments and physical activity equivalent calculations. As required by FDA regulations, calorie information was displayed for all products.^32^

### Vending Machine Sales Data

The vending machine company provided the monthly sales for each item in every machine. Sales data were provided for 9 months before the intervention (May 1, 2018, to January 31, 2019) and 13 months during the intervention (February 1, 2019, to February 29, 2020) (eAppendix 2 in Supplement 2).

### Customer Purchase Assessments and Surveys

We also collected purchases at the individual level so we could examine all beverages and snacks purchased per customer trip to determine whether there was behavioral substitution (eg, messaging may have reduced beverage calories sold but increased snack calories sold). Additionally, we wanted to examine education as a moderating variable. However, the study began on February 1, 2019, and was cut short in March 2020 by the COVID-19 pandemic, so we were unable to reach our target sample size (n = 3312), which would have enabled us to detect a 7- to 17-calorie difference across conditions by education level (eAppendix 3 in Supplement 2). To obtain the customer data, research assistants obtained verbal consent from adult customers and asked them to orally complete a brief survey after their purchase (eAppendix 3 in Supplement 2), indicating what they purchased, their vending machine habits, and perceptions of the food and beverage messaging intervention (eAppendix 3 in Supplement 2). Self-reported race and ethnicity information were collected to describe the demographics of the sample.

### Outcomes

We conducted 3 sets of analyses: 2 sets with the sales data and 1 with the customer purchase assessment data. First, at the transaction level, we assessed whether there were differences by condition in the average healthfulness of the products sold per transaction. These outcomes were preregistered: calories sold per transaction and the health status of the item sold (healthy [green], moderately healthy [yellow], or unhealthy [red]). Second, at the machine level, our preregistered outcome was the monthly number of products sold per machine. We also explored the following machine-level outcomes: total calories and number of green, yellow, and red items sold per month. We examined monthly machine revenue as a secondary outcome. Third, the primary customer purchase assessment outcome was the combined number of calories sold per trip based on the nutrition facts labels.

### Statistical Analysis

#### Vending Machine Sales Data Analyses

Beverage and snack analyses were conducted separately in an intention-to-treat framework, using Stata 16, version 16.1 (StataCorp LLC). All models used population-averaged marginal models estimated by generalized estimating equations (GEEs)^33^ with clustering by floor and independence working correlation structures to account for the correlation over time among sales on the same floor. Beverage tax was the primary reference group because it was only a poster (no labels) focused on price, not nutrition, messaging. Transaction-level beverage calorie analyses used 2-part models to assess whether condition affected selection of beverages with calories using a logistic model (because many beverages had 0 calories). Then, among beverages with calories, linear models assessed whether condition affected the number of calories sold. We used a linear model for snack calorie analyses. Multinomial logit models were used to examine the proportion of red, yellow, and green products sold, yielding relative risk ratios (RRRs) representing the proportional change in the risk of one category (eg, green) being sold compared with the risk of the referent category (red) being sold, depending on condition. Machine-level analyses used a negative binomial distribution because either they were highly right skewed or the outcome was count data. These analyses generated a mean ratio (MR), which represents the monthly mean (eg, calories or number of unhealthy items sold) in one condition compared with beverage tax.

All analyses controlled for mean monthly baseline data for the relevant outcome from the 9 months before the intervention began, vending machine location type (eg, correctional facilities), baseline machine sales (high or low), and the location type by baseline sales interaction because correctional facilities had particularly high sales compared with other location types. Models also adjusted for time (categorical month to account for seasonality in purchasing),^34,35^ the mean number of unique red items available at baseline, and whether small beverage sizes (<20 oz) were ever available during baseline. We tested for any difference across all 4 conditions at the 2-sided *P* < .05 level and then used 6 pairwise tests to probe significant effects with a Holm-Bonferroni correction for multiple comparisons. For interpretation, we present predictive margins by condition (ie, the mean prediction if everyone in the data were treated as being in that condition). We also assessed sensitivity to different modeling choices and conducted secondary analyses comparing effects in the first 3 to the last 10 months.

#### Customer Purchase Assessment Analyses

We used a marginal model fit by GEE with independence working correlation structures to examine the combined calories per visit. We included the same covariates as the sales analyses with a few exceptions (eAppendix 3 in Supplement 2). We also adjusted for potential individual confounders with greater than 10% average standardized mean difference between conditions (ie, race, educational level, and worked for the City of Philadelphia). All data analyses were performed from March 5, 2020, to November 8, 2022.

## Results

### Vending Machine Sales Data Characteristics

There were 180 686 beverages and 279 098 snacks sold in the 9-month baseline period and 259 476 beverages and 375 150 snacks sold in the 13-month intervention period. During the 22-month period, beverage sales totaled $704 540 and snack sales totaled $700 511. Mean (SD) snack item prices were lower ($1.12 [$0.28]) than nontaxed beverages ($1.38 [$0.23]) and taxed beverages ($1.76 [$0.39]). See Table 1 and eAppendix 2 in Supplement 2 for more machine details and graphs of sales over time.

**Table 1.** Characteristics of Machine Analysis Sample at Baseline^a^

Characteristic	Beverage (n = 144 machines)				Snack (n = 114 machines)			
	Beverage tax (n = 32 machines)	Green only (n = 45 machines)	Traffic light (n = 34 machines)	Physical activity (n = 33 machines)	Beverage tax (n = 24 machines)	Green only (n = 37 machines)	Traffic light (n = 26 machines)	Physical activity (n = 27 machines)
Machine location								
Correctional facilities	6 (19)	9 (20)	7 (21)	3 (9)	4 (17)	6 (16)	4 (15)	3 (11)
Courts or offices	4 (13)	6 (13)	4 (12)	3 (9)	2 (8)	4 (11)	2 (8)	2 (7)
Large offices	6 (19)	7 (16)	7 (21)	6 (18)	5 (21)	7 (19)	5 (19)	6 (22)
Police or fire stations	6 (19)	7 (16)	5 (15)	10 (30)	5 (21)	6 (16)	5 (19)	7 (26)
Recreation centers, libraries, or other	10 (31)	16 (36)	11 (32)	11 (33)	8 (33)	14 (38)	10 (38)	9 (33)
Have unhealthy items	32 (100)	43 (96)	32 (94)	33 (100)	24 (100)	35 (95)	24 (92)	27 (100)
Baseline machine information								
High sales	17 (53)	31 (69)	24 (71)	18 (55)	15 (63)	26 (70)	17 (65)	14 (52)
Unique unhealthy items available, mean (SD)	2.9 (1.2)	3.0 (1.5)	2.8 (1.3)	2.8 (1.1)	6.0 (0.4)	5.5 (1.3)	5.2 (1.6)	5.9 (0.4)
Small beverages ever available	25 (78)	34 (76)	24 (71)	30 (91)	NA	NA	NA	NA
Baseline machine-level sales, mean (SD)^b^								
Monthly sold quantity	158 (171)	159 (172)	143 (115)	92 (61)	333 (345)	314 (267)	265 (210)	167 (116)
Healthy items sold, %^c^^,^^d^	30 (8)	32 (15)	29 (12)	30 (10)	34 (5)	36 (5)	36 (6)	35 (7)
Moderately healthy items sold, %^c^^,^^d^	25 (12)	24 (14)	21 (11)	27 (13)	37 (5)	37 (4)	36 (5)	37 (6)
Unhealthy items sold, %^c^^,^^d^	45 (13)	44 (15)	49 (16)	43 (17)	29 (5)	27 (6)	28 (4)	29 (6)
Items sold have kilocalories, %^d^^,^^e^	48 (14)	46 (17)	52 (17)	46 (18)	100	100	100	100
Sold kilocalories per item if has kilocalories^d^^,^^e^	165 (48)	158 (47)	163 (56)	151 (26)	189 (9)	183 (14)	182 (12)	187 (14)
Sold kilocalories for all items^d^	81 (38)	78 (42)	87 (43)	71 (34)	189 (9)	183 (14)	182 (12)	187 (14)

Abbreviation: NA, not applicable.

^a^ Data are presented as number (percentage) unless otherwise indicated.

^b^ Baseline data come from 9 months before the intervention (May 1, 2018, to January 31, 2019).

^c^ Healthfulness of sales only assessed among machines with unhealthy (red) items available (matching analyses). Four beverage machines and 4 snack machines did not have unhealthy items available and thus are excluded from healthfulness analyses.

^d^ Healthfulness and calories of sales were only assessed among products and machines with sales that month (matching healthfulness and calorie analyses, which are conditional on sales). Three beverage machines and 5 snack machines had no sales at any point at baseline and thus are excluded from healthfulness and calorie analyses.

^e^ All snack items had calories.

### Vending Machine Sales Data Results

At the transaction level, there were no differences in the mean number of calories sold per beverage or snack transaction (Table 2). Traffic light machines were 25% more likely to sell a yellow (eg, taxed diet soda) compared with a red (eg, taxed soda) beverage compared with beverage tax machines (RRR, 1.25; 95% CI, 1.07-1.46). For snacks, green-only machines were 10% more likely to sell a green snack (eg, baked chips) compared with a red snack (eg, candy) compared with beverage tax machines (RRR, 1.10; 95% CI, 1.03-1.17). At the machine level, physical activity compared with beverage tax decreased the expected monthly number of beverages sold by 24% (MR, 0.76; 95% CI, 0.61-0.95) (Table 3). Exploratory machine-level analyses showed that physical activity specifically decreased the beverage calories sold by 35% (MR, 0.65; 95% CI, 0.50-0.86) and the number of red beverages sold by 34% (MR, 0.66; 95% CI, 0.51-0.87). Although traffic light did not show overall decreases in beverage sales, the number of red beverages sold decreased by 30% (MR, 0.70; 95% CI, 0.55-0.88). For snacks, there were no machine-level differences in sales. The 2 sensitivity analyses showed substantively similar results (eAppendix 2 in Supplement 2). Secondary analyses showed no significant pairwise differences (eAppendix 2 in Supplement 2).

**Table 2.** Transaction-Level Monthly Sales Outcomes, Conditional on Sales^a^

Condition	Has calories, OR (95% CI)^b^	Predictive margin, % with calories	Calories, if calories, b (95% CI)^b^	Predictive margin	RRR (95% CI)^c^		Predictive margins, %		
					Green (reference, red)	Yellow (reference, red)	Green	Yellow	Red
Beverages									
Beverage tax	1.0 [Reference]	51.9	1.0 [Reference]	179.2	1.0 [Reference]	1.0 [Reference]	32.6	18.9^d^	48.6
Green only	0.98 (0.88 to 1.10)	51.4	−1.69 (−6.05 to 2.67)	177.6	0.95 (0.84 to 1.06)	1.17 (1.00 to 1.36)	30.4	21.6	48.0
Traffic light	0.90 (0.81 to 1.00)	49.3	−5.07 (−10.46 to 0.31)	174.2	1.01 (0.90 to 1.14)	1.25 (1.07 to 1.46)	31.4	22.3^e^	46.3
Physical activity	0.89 (0.75 to 1.05)	49.0	−4.12 (−8.51 to 0.27)	175.1	1.05 (0.90 to 1.21)	1.33 (1.03 to 1.71)	31.7	23.1	45.3
Snacks									
Beverage tax	NA	NA	1.0 [Reference]	181.6	1.0 [Reference]	1.0 [Reference]	34.5^f^	37.5	28.0
Green only	NA	NA	0.73 (−1.73 to 3.19)	182.3	1.10 (1.03 to 1.17)	1.07 (1.00 to 1.16)	35.7^e^	37.9	26.4
Traffic light	NA	NA	0.85 (−1.92 to 3.62)	182.4	1.08 (1.02 to 1.15)	1.02 (0.94 to 1.11)	36.0	37.0	27.0
Physical activity	NA	NA	−2.05 (−5.44 to 1.33)	179.5	1.03 (0.92 to 1.14)	1.05 (0.96 to 1.15)	34.4	38.3	27.3

Abbreviations: NA, not applicable; OR, odds ratio; RRR, relative risk ratio.

^a^ Covariates in all models included time (categorical month) and several baseline machine-level characteristics: average corresponding outcome across previous 9 months, average number of unique red items available, beverages smaller than 20 oz ever available (beverage analyses only), binary sales (high vs low), 4 dummies for 5 location types, and the sales × location type interaction.

^b^ A total of 258 961 beverages were clustered on 107 locations of which 131 286 had calories; 372 376 snacks were clustered on 93 locations where there were sales.

^c^ For product healthfulness, sales in locations where unhealthy (red) items were available: 252 425 beverages were clustered on 103 locations and 357 680 snacks were clustered on 89 locations.

^d^ Indicates either which conditions differ or which conditions differ in the contrast of green (healthy), yellow (moderately healthy), or red (unhealthy), using Bonferroni-Holm adjustment for traffic light.

^e^ Indicates either which conditions differ or which conditions differ in the contrast of green (healthy), yellow (moderately healthy), or red (unhealthy), using Bonferroni-Holm adjustment for beverage tax.

^f^ Indicates either which conditions differ or which conditions differ in the contrast of green (healthy), yellow (moderately healthy), or red (unhealthy), using Bonferroni-Holm adjustment for green only.

**Table 3.** Machine-Level Monthly Sales Outcomes^a^

Condition	No. sold^b^		Calories sold^c^		No. healthy sold^c^		No. moderately healthy sold^c^		No. unhealthy sold^c^	
	Mean ratio (95% CI)	Predictive margin	Mean ratio (95% CI)	Predictive margin	Mean ratio (95% CI)	Predictive margin	Mean ratio (95% CI)	Predictive margin	Mean ratio (95% CI)	Predictive margin
Beverage machines										
Beverage tax	1.0 [Reference]	188.4^d^	1.0 [Reference]	19 870^d^	1.0 [Reference]	56.7	1.0 [Reference]	37.6	1.0 [Reference]	101.4^d^^,^^e^
Green only	0.84 (0.69-1.02)	158.1	0.81 (0.64-1.03)	16 175	0.85 (0.67-1.07)	48.2	1.10 (0.82-1.49)	41.6	0.79 (0.62-1.01)	80.6
Traffic light	0.88 (0.74-1.05)	166.0	0.74 (0.59-0.93)	14 748	0.91 (0.71-1.15)	51.3	1.05 (0.82-1.34)	39.5	0.70 (0.55-0.88)	70.5^f^
Physical activity	0.76 (0.61-0.95)	144.0^f^	0.65 (0.50-0.86)	13 006^f^	0.79 (0.61-1.03)	44.8	0.86 (0.64-1.15)	32.3	0.66 (0.51-0.87)	67.3^f^
Snack machines										
Beverage tax	1.0 [Reference]	277.4	1.0 [Reference]	46 697	1.0 [Reference]	90.3	1.0 [Reference]	96.8	1.0 [Reference]	70.3
Green only	1.06 (0.83-1.36)	295.3	1.18 (0.96-1.44)	54 881	1.22 (1.00-1.49)	110.0	1.17 (0.94-1.45)	112.9	1.17 (0.92-1.50)	82.4
Traffic light	1.09 (0.83-1.44)	303.2	1.11 (0.85-1.45)	51 895	1.18 (0.91-1.54)	107.0	1.06 (0.80-1.41)	103.1	1.29 (0.97-1.71)	90.6
Physical activity	0.98 (0.74-1.31)	272.2	1.03 (0.78-1.35)	47 890	1.01 (0.75-1.36)	91.5	1.05 (0.80-1.38)	101.6	1.00 (0.73-1.37)	70.3

^a^ Covariates in all models included time (categorical month) and several baseline machine-level characteristics: average corresponding outcome across previous 9 months, average number of unique red items available, beverages smaller than 20 oz ever available (beverage analyses only), binary sales (high vs low), 4 dummies for 5 location types, and the sales × location type interaction.

^b^ In beverage machines, 1856 clustered on 108 locations; in snack machines, 1476 clustered on 99 locations.

^c^ In beverage machines, 1817 clustered on 107 locations; in snack machines, 1411 clustered on 96 locations.

^d^ Indicates either which conditions differ or which conditions differ in the contrast of green (healthy), yellow (moderately healthy), or red (unhealthy), using Bonferroni-Holm adjustment for physical activity.

^e^ Indicates either which conditions differ or which conditions differ in the contrast of green (healthy), yellow (moderately healthy), or red (unhealthy), using Bonferroni-Holm adjustment for traffic light.

^f^ Indicates either which conditions differ or which conditions differ in the contrast of green (healthy), yellow (moderately healthy), or red (unhealthy), using Bonferroni-Holm adjustment for beverage tax.

### Customer Purchase Assessment Results

A total of 1065 participants completed the purchase assessments. An additional 604 individuals declined to participate, and 164 were excluded as repeat customers, resulting in a 67% response rate. Of the participants who completed the customer purchase assessments and provided self-identified data, 558 (52%) were male and 482 (45%) were female; 24 (2%) were Asian, 556 (52%) were Black or African American, 368 (35%) were White, 79 (7%) were other race (with individuals providing various responses, such as “mixed race,” “Italian,” “Hispanic,” and “human”), and 38 (4%) refused to report or did not know race; 92 (9%) were Hispanic, 941 (88%) were non-Hispanic, and 32 (3%) refused to report or did not know ethnicity; 536 (50%) were younger than 45 years; 559 (52%) had a college degree or greater education; 456 (43%) had an annual household income of $75 000 or less; and 922 (87%) worked for the city of Philadelphia (Table 4). Of the 1065 participants, we excluded 55 participants (5%) missing at least 1 demographic covariate, leaving 1010 participants in the analytic sample.

**Table 4.** Customer Demographics From Purchase Assessments^a^

Demographic	No. (%) of participants				
	Overall (N = 1065)	Beverage tax (n = 256)	Green only (n = 335)	Traffic light (n = 246)	Physical activity (n = 228)
Gender					
Male	558 (52)	135 (53)	178 (53)	131 (53)	114 (50)
Female	482 (45)	115 (45)	151 (45)	107 (44)	109 (48)
Other	3 (0.3)	1 (0.4)	0	2 (0.8)	0
Refused	22 (2)	5 (2)	6 (2)	6 (3)	5 (2)
Race					
Asian	24 (2)	6 (2)	8 (2)	8 (3)	2 (1)
Black or African American	556 (52)	137 (54)	166 (50)	128 (52)	125 (55)
White	368 (35)	88 (34)	120 (36)	80 (33)	80 (35)
Other^b^	79 (7)	17 (7)	28 (8)	23 (9)	11 (5)
Refused or did not know	38 (4)	8 (3)	13 (4)	7 (3)	10 (4)
Ethnicity					
Hispanic	92 (9)	18 (7)	34 (10)	25 (10)	15 (7)
Not Hispanic	941 (88)	232 (91)	289 (86)	213 (87)	207 (91)
Refused or did not know	32 (3)	6 (2)	12 (4)	8 (3)	6 (3)
Age group, y					
18-24	55 (5)	9 (4)	21 (6)	15 (6)	10 (4)
25-34	229 (22)	50 (20)	65 (19)	61 (25)	53 (23)
35-44	252 (24)	67 (26)	80 (24)	60 (24)	45 (20)
45-54	271 (25)	58 (23)	93 (28)	54 (22)	66 (29)
55-64	175 (16)	51 (20)	51 (15)	40 (16)	33 (14)
≥65	32 (3)	6 (2)	11 (3)	9 (4)	6 (3)
Refused	51 (5)	15 (6)	14 (4)	7 (3)	15 (7)
Educational level					
High school or less	220 (21)	50 (20)	67 (20)	45 (18)	58 (25)
Some college or associate’s degree	257 (24)	52 (20)	87 (26)	70 (28)	48 (21)
College or higher	559 (52)	149 (58)	172 (51)	125 (51)	113 (50)
Refused	29 (3)	5 (2)	9 (3)	6 (2)	9 (4)
Marital status					
Married or living with partner	432 (41)	101 (39)	139 (41)	93 (38)	99 (43)
Single	583 (55)	140 (55)	186 (56)	142 (58)	115 (50)
Refused	50 (5)	15 (6)	10 (3)	11 (4)	14 (6)
Annual household income, $					
≤25 000	37 (3)	6 (2)	12 (4)	11 (4)	8 (4)
25 001-50 000	180 (17)	36 (14)	50 (15)	51 (21)	43 (19)
50 001-75 000	239 (22)	58 (23)	82 (24)	56 (23)	43 (19)
75 001-100 000	187 (18)	50 (20)	60 (18)	42 (17)	35 (15)
100 001-125 000	80 (8)	13 (5)	29 (9)	19 (8)	19 (8)
125 001-150 000	55 (5)	13 (5)	17 (5)	14 (6)	11 (5)
>150 000	89 (8)	25 (10)	27 (8)	18 (7)	19 (8)
Refused	198 (19)	55 (21)	58 (17)	35 (14)	50 (22)
BMI					
Underweight	8 (1)	1 (0.4)	6 (2)	1 (0.4)	0
Healthy weight	206 (19)	50 (20)	58 (17)	57 (23)	41 (18)
Overweight	306 (29)	73 (29)	93 (28)	74 (30)	66 (29)
Obesity	381 (36)	96 (38)	118 (35)	88 (36)	79 (35)
Refused or did not know	164 (15)	36 (14)	60 (18)	26 (11)	42 (18)
Work for the city in any way					
Yes	922 (87)	232 (91)	291 (87)	206 (84)	193 (85)
No	114 (11)	18 (7)	35 (10)	35 (14)	26 (11)
Refused or did not known	29 (3)	6 (2)	9 (3)	5 (2)	9 (4)

Abbreviation: BMI, body mass index (calculated as weight in kilograms divided by height in meters squared).

^a^ Due to rounding, percentages do not always total 100%.

^b^ Other includes individuals who provided various responses, such as “mixed race,” “Italian,” “Hispanic,” and “human.”

Traffic light labels significantly decreased total calories sold per customer trip compared with physical activity (147 vs 178 kcal; b = −30.46; 95% CI, −49.36 to −11.56) and nonsignificantly decreased total calories compared with beverage tax after Bonferroni-Holm adjustment (180 kcal) (eAppendix 3 in Supplement 2). Sensitivity analyses showed similar results. Education did not significantly moderate effects (eAppendix 3 in Supplement 2).

Only 65 participants (26%) in the beverage tax group reported noticing “any posters or labels displayed on the machines” compared with 133 (40%) in the green-only group (odds ratio [OR], 1.90; 95% CI, 1.33-2.72), 131 (58%) in the physical activity group (OR, 3.93; 95% CI, 2.67-5.78), and 161 (65%) in the traffic light group (OR, 5.45; 95% CI, 3.71-8.01). Reported noticing of messaging did not moderate condition effects on calories sold per trip.

## Discussion

In this large randomized trial directly comparing 4 types of food and beverage messages, we found that traffic light labels and physical activity calorie equivalent labels were more successful at encouraging healthy beverage purchasing than posters reminding customers of Philadelphia’s sweetened beverage tax. Green-only labeling did not significantly differ from beverage tax posters. These results are consistent with a laboratory study that found presenting front-of-package healthy or unhealthy labels on all items led to healthier beverage and snack purchases compared with labels on only some items.^36^ Finally, we did not see large changes in snack sales. The lack of elasticity in snack purchasing could be because the price differential between available healthy and unhealthy snacks was much smaller than the differential between beverages.

Physical activity labels on beverages decreased monthly calories, unhealthy beverages, and overall beverages sold, indicating that some customers stopped buying unhealthy beverages in response to the labels rather than switching to healthier alternatives. This interpretation is consistent with no significant transaction-level differences in beverage calories sold and a field experiment comparing physical activity equivalent labels to baseline sales.^9^

Traffic light labels decreased machine-level unhealthy beverage sales. There were no significant differences in overall beverage sales and fewer sales of unhealthy items compared with moderately healthy items at the transaction level, suggesting that the overall reduction in unhealthy beverage sales may have been driven by people switching to healthier beverages (eg, regular to diet soda, both of which were taxed, so pricing was the same). These results are consistent with a cafeteria study of traffic light labeling where purchases of unhealthy beverages decreased more than healthy beverages.^7^ They differ from a labeling meta-analysis, however, which found that traffic light labeling primarily increased healthy purchases rather than decreasing less healthy purchases.^37^ However, most of those studies were laboratory based. Among the customer purchase assessments, traffic light labels also decreased the calories sold per trip, suggesting customers did not buy unhealthy snacks when shifting to healthier beverages.

Secondary analyses showed no differences. Intervention effects were the same in the first 3 months as the last 10 months of the study. Despite physical activity labels decreasing overall beverage sales during the study, monthly dollar sales for beverage and snack machines did not decrease in any condition. However, in the sensitivity analysis eliminating potentially cumulative data, physical activity labels decreased monthly dollar sales compared with beverage tax posters.

### Strengths and Limitations

This study has several strengths. First, we compared multiple food and beverage messages using a randomized field experiment in a large number of vending machines. Second, these machines were in a diverse range of settings rather than concentrated in a single setting (eg, worksite) as in other studies.^38^ Third, we studied our intervention in both beverage and snack machines and for a long period. Fourth, we evaluated objectively measured machine-level sales data and customer purchase assessments.

This study also has several limitations. First, it was conducted in vending machines that were subject to city regulations that made them healthier than average, so results may not generalize to other vending machines or purchasing settings. Second, because the city required that all vending machines have some type of label for healthy items, we were unable to test a pure control group with no labeling intervention. Instead, beverage tax posters provided an interesting comparison group. Our test of tax salience messaging may have been diluted because some beverage tax posters fell off. Only one-quarter of participants reported noticing the tax reminders compared with 58% and 65% noticing the physical activity and traffic light messaging, respectively. There were differences in baseline sales by condition, although we adjusted for these. Still, customers in the physical activity locations (which had lower baseline sales) may have been less frequent vending consumers. Our customer response rate was 67%, which is higher than for studies recruiting outside urban food retailers.^39^ Finally, the start of the COVID-19 pandemic prevented us from reaching our target customer sample size.

## Conclusions

This 13-month randomized trial of 267 vending machines demonstrated that traffic light and physical activity calorie equivalent labels encouraged healthier beverage, but not snack, purchases compared with a beverage tax poster by leading customers to buy nothing or choose a healthier alternative. Corporations and governments should consider such labeling approaches to promote healthier beverage choices.

## Notes

Supplement 1.
Trial Protocol

Supplement 2. **eAppendix 1.** Treatment Information **eAppendix 2.** Sales Data **eAppendix 3.** Customer Purchase Assessments

Supplement 3.
Data Sharing Statement
