# Supplementary material for: Comparison of Sales From Vending Machines With 4 Different Food and Beverage Messages: A Randomized Trial
Source: JAMA Netw Open. 2024 May 8;7(5):e249438. doi: 10.1001/jamanetworkopen.2024.9438 (PMC11079689; doi:10.1001/jamanetworkopen.2024.9438)
Supplement: Supplement 1. — Trial Protocol [file jamanetwopen-e249438-s001.pdf]

Comparison of Sales from Vending Machines with 4 Different Food and Beverage Messages: A Randomized Field Experiment Trial Protocol and Analysis Plan

Notes

Per journal instructions, this document contains the trial protocol and analysis plan as it was submitted to the University of Pennsylvania Institutional Review Board. We additionally pre-registered the analysis plan through AsPredicted (https://aspredicted.org/G5X_WSS). Deviations from this plan are detailed and justified in the main text.

Protocol

Objectives

Overall objectives

Aim 1: To compare the influence of four nutrition messages on the average calories and percent of healthy beverages purchased over three years.
Aim 2: To compare the influence of three nutrition messages on the average calories and percent of healthy snacks purchased over three years.
Aim 3: To examine the degree to which education level moderates the influence of nutrition messaging on average calories and percent of healthy foods and beverage purchased.

Background

Poor dietary habits are a significant public health problem. Unhealthy dietary habits are associated with obesity, cardiovascular disease, type 2 diabetes, and certain cancers (1). To comprehensively address these chronic diseases, interventions need to focus on calorie reduction and changing specific dietary habits. For example, potato chips and sweetened beverages are two foods with the strongest links to long-term weight gain, suggesting a need to focus on decreasing their consumption, while vegetables, whole grains, fruits, nuts, and yogurt are protective against weight gain (2). This project aims to determine which nutrition messages are best able to encourage lower calorie and higher nutritional quality food choices.

Consumption at vending machines. U.S. sales from food and beverage vending machines exceed $11.7 billion yearly (3). Although the goal of this study is to provide a rigorous evaluation of nutrition messaging interventions more broadly, there is reason to care specifically about altering vending machine purchases. Altering snacking behavior at vending machines has the potential to impact weight. Researchers have estimated a weight maintenance energy gap, which is the energy needed to stop gaining additional weight and to lose excess weight. If we want to return to 1970 levels of excess weight in the population, it is estimated that the average adult would have to consume 240 fewer calories per day (4). This highlights the benefits of helping consumers switch from a high calorie vending snack (e.g., a 400 calorie bag of cookies) several times a week to a lower calorie snack (e.g., 150 calorie bag of popcorn).

Despite the prevalence of vending machine snacking, only a handful of studies have assessed labeling interventions in vending machines and none have compared multiple, newer labeling systems using a randomized design with a large sample of machines and long-term follow up. Further, the majority of studies have only tested low-calorie or low-fat labels, rather than newer labeling proposals, such as traffic lights, designed to make information more meaningful and salient. Overall, research on labeling effects in vending machines is mixed. There have been four nonrandomized vending studies. One study of 7 vending machines in an office found no purchasing effect from labeling snacks as low-calorie over 14 months and another study of 4 vending machines found no purchasing effect of calorie labels plus percent daily recommended amounts for certain nutrients (5,6). One study testing a multiple traffic light label in 5 university vending machines over 4 weeks found a 51% increase in healthy items from baseline to post intervention, although one machine showed significant declines in green-stickered items sold (7). Another study of traffic lights over 28 weeks in 9 state agency vending machines that also required 75% of items to be healthy found that the proportion of healthy items sold increased at some locations and decreased at others (8).

There have only been four randomized vending label studies. In a 12-month intervention, French and colleagues randomly assigned 55 vending machines in multiple settings (i.e. secondary schools and worksites) to a 4 arm pricing x 3 arm label design (9). Low-fat labels alone had no effect, but combining them with promotional signage led to increased sales of low-fat snacks. Similarly, an 8-week study randomizing 8 vending machines on an urban college campus to either calorie labels or zero-calorie labels on beverages plus motivational signs to purchase zero calorie drinks found that only labels plus signage resulted in a significantly lower growth rate of sugary drink sales compared to the control arm (10). In

contrast, Fiske and Cullen conducted a 6-week, randomized-controlled trial of 10 vending machines in teachers lounges of elementary and middles schools and found no impact of yellow labels highlighting price stickers for low-fat items or signage for low-fat snacks (11). A final randomized-controlled trial of 18 machines studied over 16 weeks in university resident halls found no purchasing effect of better choice labels placed on healthy foods combined with nearby posters displaying nutrition facts labels (12).

Education may be an important moderator of nutrition message effects. One worry with nutrition labeling interventions is that such strategies only influence those with higher education levels. For example, more educated people are much more likely to report using the nutrition facts label on packaged foods (13,14). However, the hope is that easier-to-understand nutrition messages such as traffic light labels will have equal impact across the education gradient. The proposed research is designed and powered to examine potential differences in label influence based on education level because education is a direct mechanism through which nutrition labels can influence behavior, as opposed to other demographic characteristics that are predictive of label use (e.g., gender), but are not sufficient to explain why an individual is using a nutrition label. In summary, we currently lack rigorous, randomized, long-term evaluations with objective purchasing data to compare the degree to which different novel nutrition messages influence behavior and whether those across education levels are equally impacted by these messages.

1. Mozaffarian D. Dietary and policy priorities for cardiovascular disease, diabetes, and obesity: a comprehensive review. Circulation. 2016;133 (2):187-225.
2. Mozaffarian D, Hao T, Rimm EB, Willett WC, Hu FB. Changes in diet and lifestyle and long-term weight gain in women and men. N Engl J Med. 2011;364(25):2392-2404.
3. Packaged Facts. Food and Beverage Vending Trends in the U.S. 2013.
4. Gortmaker SL, Swinburn BA, Levy D, et al. Changing the future of obesity: science, policy, and action. Lancet. 2011;378(9793):838-847.
5. Wilbur CS, Zifferblatt SM, Pinsky JL, Zifferblatt S. Healthy vending: a cooperative pilot research program to stimulate good health in the marketplace. Prev Med. 1981;10(1):85-93.
6. Hoerr SM, Louden VA. Can nutrition information increase sales of healthful vended snacks? J Sch Health. 1993;63:386390.
7. Brown MV, Flint M, Fuqua J. The effects of a nutrition education intervention on vending machine sales on a university campus. J Am Coll Health. 2014;62(7):512-516.
8. Lessard L, Poland M, Trotter M. Lessons learned from a healthful vending pilot program in Delaware state agency buildings, 2011-2012. Prev Chronic Dis. 2014;11:E143.
9. French SA, Jeffery RW, Story M, et al. Pricing and promotion effects on low-fat vending snack purchases: the CHIPS Study. Am J Public Health. 2001;91 (1):112-117.
10. Bergen D, Yeh MC. Effects of energy-content labels and motivational posters on sales of sugar-sweetened beverages: stimulating sales of diet drinks among adults study. J Am Diet Assoc. 2006;106(11):1866-1869
11. Fiske A, Cullen KW. Effects of promotional materials on vending sales of low-fat items in teachers lounges. J Am Diet Assoc. 2004;104(1):90-93.
12. Dingman DA, Schulz MR, Wyrick DL, Bibeau DL, Gupta SN. Does providing nutrition information at vending machines reduce calories per item sold? J Public Health Policy. 2015;36(1):110-22.
13. Campos S, Doxey J, Hammond D. Nutrition labels on pre-packaged foods: a systematic review. Public Health Nutr. 2011;14 (8):1496-1506.
14. Malam S, Clegg S, Kirwan S, et al. Comprehension and use of UK nutrition signpost labelling schemes. London: Food Standards Agency;2009.

Study Design

Design

The city of Philadelphia has 354 vending machines on property owned or leased by the City. We have a list of all the machines and have excluded 89 low-performing machines that have lower than $2000 in sales per year. This left 160 beverage and 107 snack vending machines. Machine randomization will be stratified based on location and sales performance. The current breakdown of beverage vending machine locations is as follows: large office buildings (23%), police and fire stations/offices (22%), correctional facilities (19%), municipal courts and associated offices (12%), recreation centers and libraries (6%), and other city owned property (18%) such as parking garages and utility facilities. The breakdown of snack vending machine locations is as follows: large office buildings (25%), correctional facilities (21%), police and fire stations/offices (19%), other city owned property (19%) such as parking garages and utility facilities, municipal courts and associated offices (13%), and recreation centers and libraries (4%).

All vending machines will display calorie labels as required by the current City vending contract. Snack vending machines will be randomized to one of three additional nutrition labeling interventions: 1) single green traffic light labels; 2) multiple traffic light labels; or 3) physical activity labels. Beverage vending machines will have an additional randomized arm to test messaging about the Philadelphia beverage tax. (See Appendix C for labels and posters.) On each vending machine, we will also display an instructional poster explaining what each label signifies. We will design the posters to provide the minimum instruction needed to understand each labeling system without additional persuasive messaging. For example, in the physical activity label condition, the poster will read: Did you know it takes 110 minutes of brisk walking to work off a chocolate bar? while the traffic light poster will say, Healthy Snack Guide: Look for snacks with green labels for a healthier choice.

We will first randomize beverage vending machines stratified by sales performance and location type. We will ensure machines are clustered at the floor level to avoid spillover effects so that all machines on the same floor display the same label. Snack vending machines will then be assigned the same intervention arm as the beverage machines on the same floor to avoid spillover effects (except for tax messaging, which does not apply to snack machines). The remaining snack machines will then be randomized (also stratified based on location and sales performance) to either: 1) single green traffic lights; 2) multiple traffic lights or 3) physical activity labels. Appendix D describes the messaging arms and nutrition criteria for labeling products. See Appendix C for labels and posters.

Research assistants will conduct audits once per quarter during each year of data collection to ensure machines are getting the appropriate labels and posters for its randomization assignment (See Appendix E for audit form). Trained research assistants will also survey 1,656 beverage vending machine patrons (~414 people per label condition) and 1,260 snack vending machine patrons (~420 people per label condition) by intercepting them after they make a purchase at a vending machine (See Appendix B for survey). We will aim to recruit 10 people per beverage machine and 11 people per snack machine. Potential participants will be asked if they would be interested in participating in a 5-minute survey for $5 gift card. We will begin these surveys after the messages have been in place for 3 months to ensure effects are not solely driven by novelty. Participants will be asked whether they previously completed a similar survey and if so, will not be eligible to participate again. We will also record the number of individuals who decline to participate and their approximate age and gender to assess whether participants differ from non-participants.

Study duration

We anticipate this will be a four-year study. It will take approximately one month to put up all the labels and posters on the vending machines. These labels and posters will stay up for three years, and we will receive monthly sales reports from the vendors. We project it will take three years to collect all the customer intercept surveys (each survey will take approximately five minutes to administer). From there, it will take another 6-8 months to analyze the data and write up the results. We will roll out the study once we receive IRB approval.

Characteristics of the Study Population

Target population

Any person aged 18 or older making a purchase from the vending machines included in this intervention will be asked to participate in this study. People who indicate they have already taken the survey are not eligible to take the survey again.

Subjects enrolled by Penn Researchers

2,916

Subjects enrolled by Collaborating Researchers

0

Vulnerable Populations

Children Form

Pregnant women (if the study procedures may affect the condition of the pregnant woman or fetus) Form

Fetuses and/or Neonates Form

Prisoners Form

Other

x **None of the above populations are included in the research study**

The following documents are currently attached to this item:

There are no documents attached for this item.

Subject recruitment

Trained research assistants standing by the vending machines will ask potential participants if they are interested in completing a short survey about their vending machine purchase for a $5 gift card. They will be asked whether they have previously completed a similar survey and if so, will not be eligible to participate again. We will also record the number of individuals who decline to participate and their approximate age and gender to assess whether participants differ from non-participants.

Will the recruitment plan propose to use any Penn media services (communications, marketing, etc.) for outreach via social media avenues (examples include: Facebook, Twitter, blogging, texting, etc.) or does the study team plan to directly use social media to recruit for the research?

No

The following documents are currently attached to this item:

There are no documents attached for this item.

Subject compensation*

Will subjects be financially compensated for their participation?

Yes

The following documents are currently attached to this item:

There are no documents attached for this item.

If there is subject compensation, provide the schedule for compensation per study visit or session and total amount for entire participation, either as text or separate document

Participants will be offered a $5 gift card for their time.

Study Procedures

Suicidal Ideation and Behavior

Does this research qualify as a clinical investigation that will utilize a test article (ie- drug or biological) which may carry a potential for central nervous system (CNS) effect(s)?

No

Procedures

The city of Philadelphia has 354 vending machines on property owned or leased by the City. We have a list of all the machines and have excluded 89 low-performing machines that have lower than $2000 in sales per year. This left 160 beverage and 107 snack vending machines. Machine randomization will be stratified based on location and sales performance. The current breakdown of beverage vending machine locations is as follows: large office buildings (23%), police and fire stations/offices (22%), correctional facilities (19%), municipal courts and associated offices (12%), recreation centers and libraries (6%), and other city owned property (18%) such as parking garages and utility facilities. The breakdown of snack vending machine locations is as follows: large office buildings (25%), correctional facilities (21%), police and fire stations/offices (19%), other city owned property (19%) such as parking garages and utility facilities, municipal courts and associated offices (13%), and recreation centers and libraries (4%). All vending machines will display calorie labels as required by the current City vending contract. Snack vending machines will be randomized to one of three additional nutrition labeling interventions: 1) single green traffic light labels; 2) multiple traffic light labels; or 3) physical activity labels. Beverage vending machines will have an additional randomized arm to test messaging about the Philadelphia beverage tax. (See Appendix C for labels and posters.) On each vending machine, we will also display an instructional poster explaining what each label signifies. We will design the posters to provide the minimum instruction needed to understand each labeling system without additional persuasive messaging. For example, in the physical activity label condition, the poster will read: Did you know it takes 110 minutes of brisk walking to work off a chocolate bar? while the traffic light poster might say, Healthy Snack Guide: Look for snacks with green labels for a healthier choice. We will first randomize beverage vending machines stratified by sales performance and location type. We will ensure machines are clustered at the floor level to avoid spillover effects so that all machines on the same floor display the same label. Snack vending machines will then be assigned the same intervention arm as the beverage machines on the same floor to avoid spillover effects (except for tax messaging, which does not apply to snack machines). The remaining snack machines will then be randomized (also stratified based on location and sales performance) to either: 1) single green traffic lights; 2) multiple

traffic lights or 3) physical activity labels. Appendix D describes the messaging arms and nutrition criteria for labeling products. Research assistants will conduct audits once per quarter during each year of data collection to ensure machines are getting the appropriate labels and posters for its randomization assignment (See Appendix E for audit form). Trained research assistants will also survey 1,656 beverage vending machine patrons (~414 people per label condition) and 1,260 snack vending machine patrons (~420 people per label condition) by intercepting them after they make a purchase at a vending machine (See Appendix B for survey). We will aim to recruit 10 people per beverage machine and 11 people per snack machine. Potential participants will be asked if they would be interested in participating in a 5-minute survey for $5 gift card. We will begin these surveys after the messages have been in place for 3 months to ensure effects are not solely driven by novelty. Participants will be asked whether they previously completed a similar survey and if so, will not be eligible to participate again. We will also record the number of individuals who decline to participate and their approximate age and gender to assess whether participants differ from non-participants. All participants will be given a Participant Information Sheet outlining the details of the study. See Appendix A.

The following documents are currently attached to this item:

Procedures (appendixa-participantinformationsheetirb.doc)
Procedures (appendixb-vendinginterceptsurveyirb.docx)
Procedures (appendixc-labelsandpostersirb.pdf)
Procedures (appendixd-nutritionmessagecriteriairb.docx)
Procedures (appendixe-vendingmachinesauditirb.docx)

Deception

Does your project use deception?
No

International Research

Are you conducting research outside of the United States?
No

Analysis Plan

<u>Analysis of Nutrition Labels</u>: Data will be descriptively summarized and evaluated for quality prior to the evaluation of primary and secondary endpoints. Means and standard deviations will be used to characterize continuous variables such as calories purchased, and frequencies and percentages will be used to describe categorical variables such as nutritional quality. Medians and interquartile ranges will be reported for continuous variables that exhibit skewness. Data will be descriptively summarized overall and by messaging strategy.

All analyses will be stratified by snack or beverage machine, and all outcomes will be analyzed at the level of the item purchased. Analyses will employ population-averaged marginal models estimated by Generalized Estimating Equations (GEE) to account for correlation among purchases within the same machine in a given month and longitudinally across months. GEE will also be used to handle clustering of beverage machines within location. Independence working correlation will be assumed for all analyses. Marginal models estimated by GEE are advantageous for their population-level interpretation and robustness of estimated effects to the assumed correlation structure among repeated measurements (meaning unbiased estimates of the messaging strategy will be obtained whether or not the correlation among repeated measurements with a vending machine is modeled correctly). Proportional odds models will be used for the ordinal outcome of healthy, moderately healthy, and less healthy items sold, and linear models will be used for the continuous outcome of calories per item sold. Models will include nutrition message intervention arm, month, and the stratification variables location and sales performance level. If data show evidence of lack of proportionality respective baseline category logit models will be used. These models allow for the estimation of effects on outcomes of 3 categories without relying on the proportional odds assumption. To increase precision, we will also adjust for baseline average calories purchased and baseline percent of healthy items purchased when analyzing differences in average calories and percent of healthy items purchased, respectively. Month will be entered into the model as a continuous variable. A quadratic month variable will be considered to flexibly model underlying trends in changes in items purchased and offered. If not significant at the 0.05 level, the quadratic terms will be removed to enhance interpretability.

For beverage machines, we will first conduct an overall test of any difference in mean calories or percent of healthy items purchased across all 4 arms (tax message, physical activity, multiple traffic light, and single green light) at the 0.05 level. If data provide evidence of differences, subsequent pairwise tests will be conducted. To account for multiple comparisons

of intervention arms we will use the Holm-Bonferroni method.67 Similarly, among snack machines we will first conduct an overall test of differences across all 3 arms (physical activity, multiple traffic light, and single green light) in mean calories and percent of healthy items purchased. Secondary analyses will assess changes in intervention effects over time by evaluating the interaction of intervention and month variables. Interactions will be evaluated by tests of the null hypothesis that interaction coefficients are 0. We will evaluate the sensitivity of estimated intervention effects to missing data. To assess the impact of imbalance in baseline covariates, outcomes will be reanalyzed in models that further adjust for baseline covariates that differ significantly at the 0.05 level by labeling intervention. We do not expect substantial missing data due to the supply of data by vending machine vendors. However, we will evaluate sensitivity to missing data by inverse probability of weighted GEE and multiple imputation.

<u>Analysis of Intercept Surveys</u>: For this cross-sectional study, the unit of analysis is the individual consumer, with clustering of individuals within vending machine. All analyses will be stratified by snack or beverage machine. Participant characteristics will be descriptively summarized overall and by intervention arm prior to analysis. Means and standard deviations will be used for continuous variables such as age, and frequencies and percentages will be used for categorical variables such as gender and education level. Population-averaged marginal models estimated by Generalized Estimating Equations will be used to account for correlation in responses among individuals using the same vending machine. A linear model will be used for the continuous outcome of average calories and a proportional odds model will be used for the ordinal outcome of healthy, moderately healthy, or less healthy item purchased. Models will include intervention arm, the potential effect modifier (e.g., education level), and their interaction, and an independence correlation structure will be assumed for estimation. For snack machines, we will first assess the interaction of education level with a dichotomous intervention variable that indicates the single traffic light labeling system versus the multiple traffic light and physical activity labels. This one degree-of-freedom test maximizes power for tests of interactions under our hypothesis of similar behavior between demographic groups using machines in the multiple traffic light and physical activity label groups and different behavior between demographic groups using machines in the single traffic light label group. The presence of an interaction will be evaluated by the two-sided test of the null hypothesis that the interaction coefficient is 0. Tests of interactions will be conducted at the 0.05 level. As a secondary analysis, we will also estimate interaction effects for the three-level intervention label strategy with demographic variables. We will similarly first assess interactions of demographic characteristics with a dichotomous intervention variable for single traffic light versus tax label, physical activity, or multiple traffic light interventions and continue with interactions of the four-level intervention variable for beverage machines.
