# Supplementary material for: Comparison of Sales From Vending Machines With 4 Different Food and Beverage Messages: A Randomized Trial
Source: JAMA Netw Open. 2024 May 8;7(5):e249438. doi: 10.1001/jamanetworkopen.2024.9438 (PMC11079689; doi:10.1001/jamanetworkopen.2024.9438)
Supplement: Supplement 2. — eAppendix 1. Treatment Information eAppendix 2. Sales Data eAppendix 3. Customer Purchase Assessments [file jamanetwopen-e249438-s002.pdf]

Supplementary Online Content

Gibson LA, Stephens-Shields AJ, Hua SV, et al. Comparison of sales from vending machines with 4 different food and beverage messages: a randomized trial. *JAMA Netw Open*. 2024;7(5):e249438. doi:10.1001/jamanetworkopen.2024.9438

eAppendix 1. Treatment Information
eAppendix 2. Sales Data
eAppendix 3. Customer Purchase Assessments

This supplementary material has been provided by the authors to give readers additional information about their work.

eAppendix 1. Treatment Information

A1. Labels and Posters

1. Beverage tax. The beverage poster was developed by the City of Philadelphia. We adapted it to make the snack poster.

Beverage Labels
[N/A]

Snack Labels
[N/A]

Beverage Poster (hang tag)

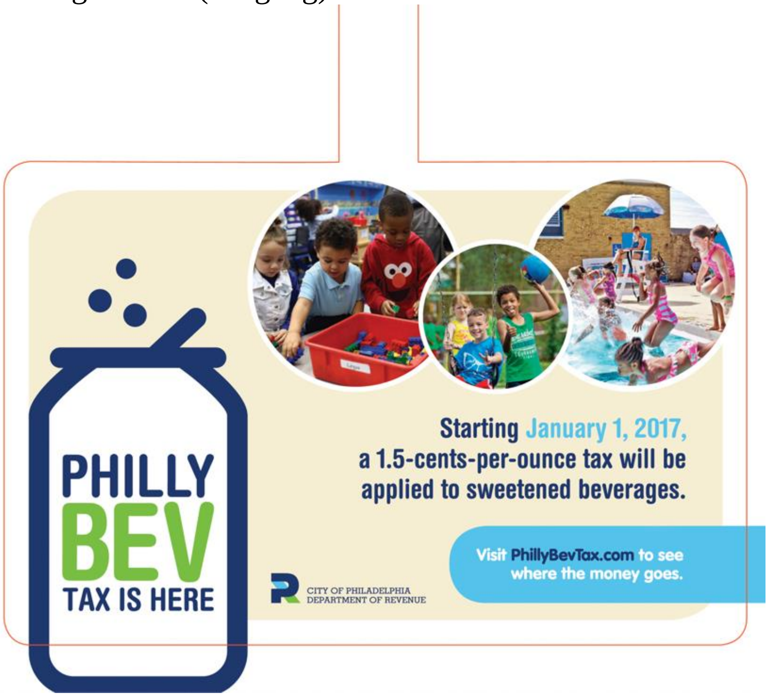

Snack Poster

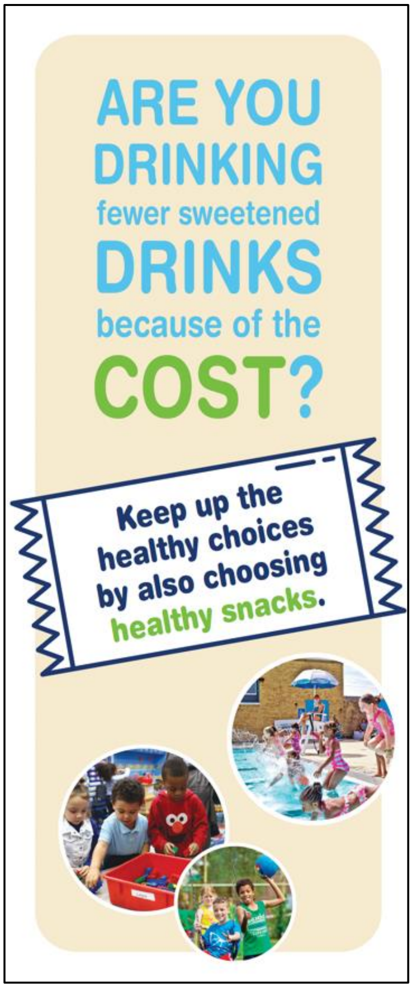

© 2024 Gibson LA et al. *JAMA Network Open.*

2. Green only. These were based on the "green" condition labels and posters from Thorndike and colleagues.[1]

Beverage Labels

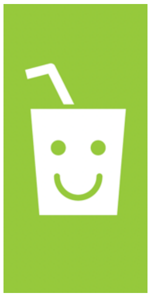

Snack Labels

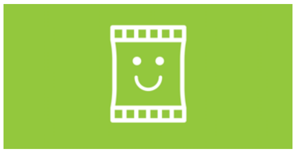

Beverage Poster

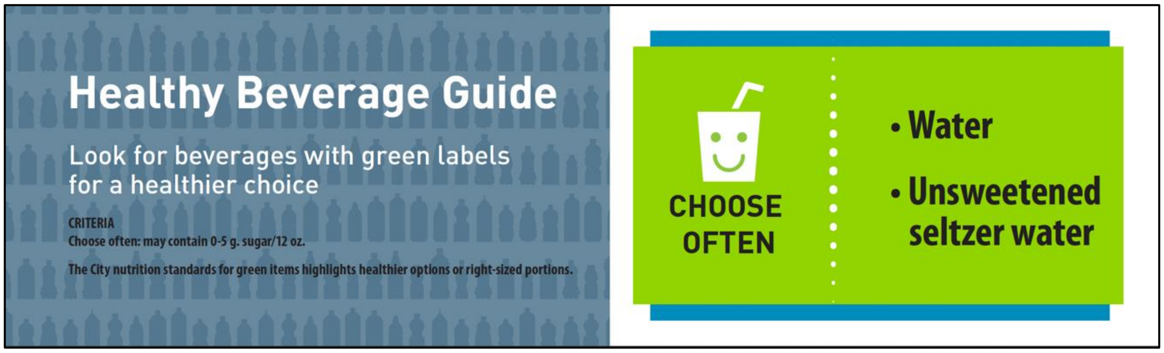

Snack Poster

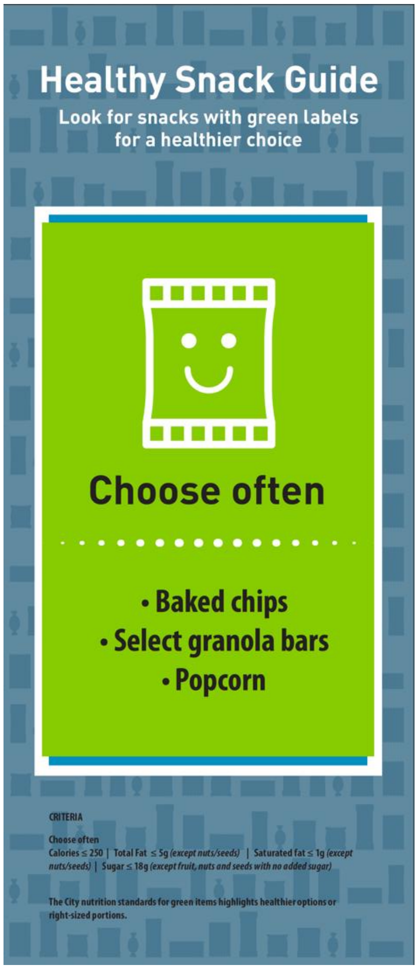

[1] Thorndike AN, Riis J, Sonnenberg LM, Levy DE. Traffic-light labels and choice architecture: promoting healthy food choices. *Am J Prev Med*. 2014;46(2):143-149.

© 2024 Gibson LA et al. *JAMA Network Open.*

3. Traffic light. These were based on the labels and posters from Thorndike and colleagues.[2]

Beverage Labels

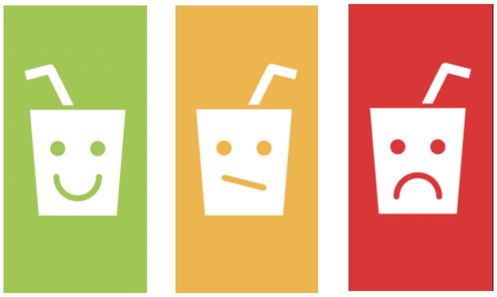

Snack Labels

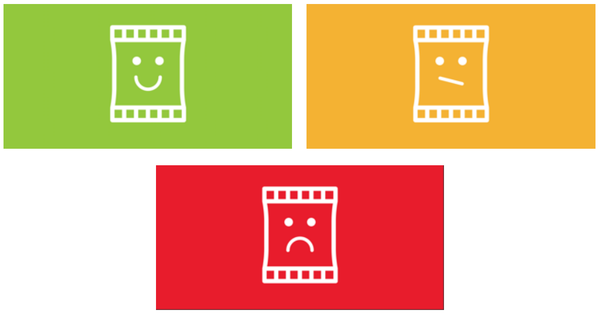

Beverage Poster

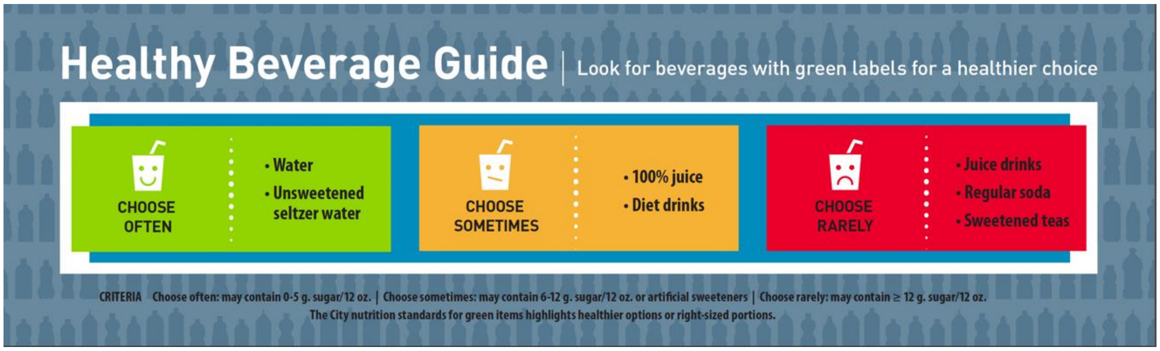

Snack Poster

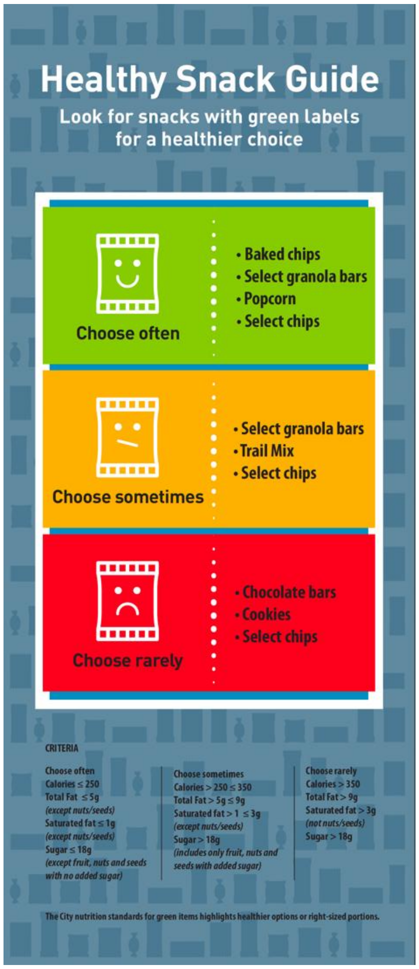

[2] Thorndike AN, Riis J, Sonnenberg LM, Levy DE. Traffic-light labels and choice architecture: promoting healthy food choices. *Am J Prev Med*. 2014;46(2):143-149.

© 2024 Gibson LA et al. *JAMA Network Open.*

4. Physical activity. These were based on the labels and posters from Bleich and colleagues.[3]

Beverage Labels

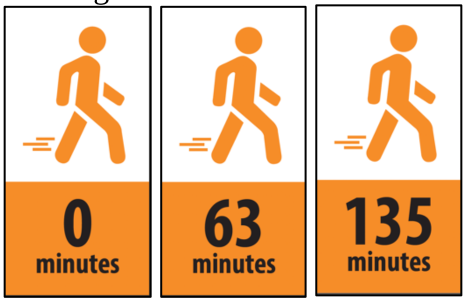

Snack Labels

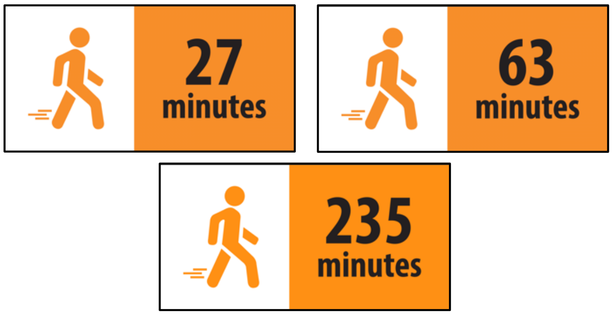

Beverage Poster

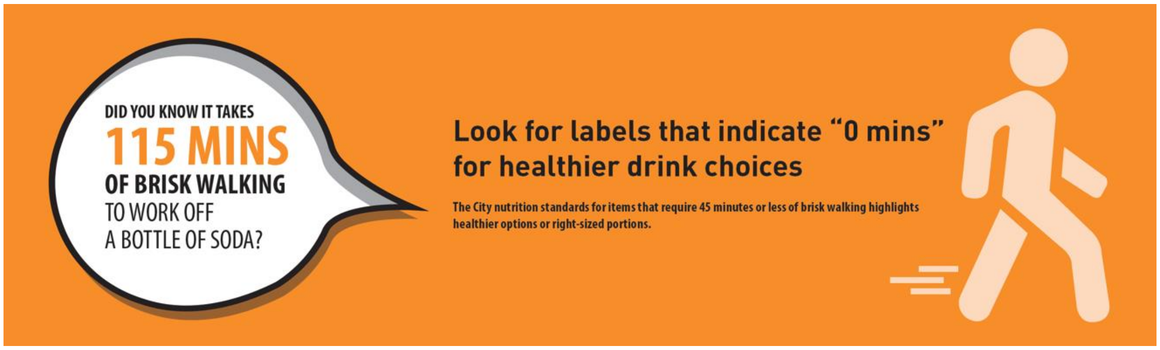

Snack Poster

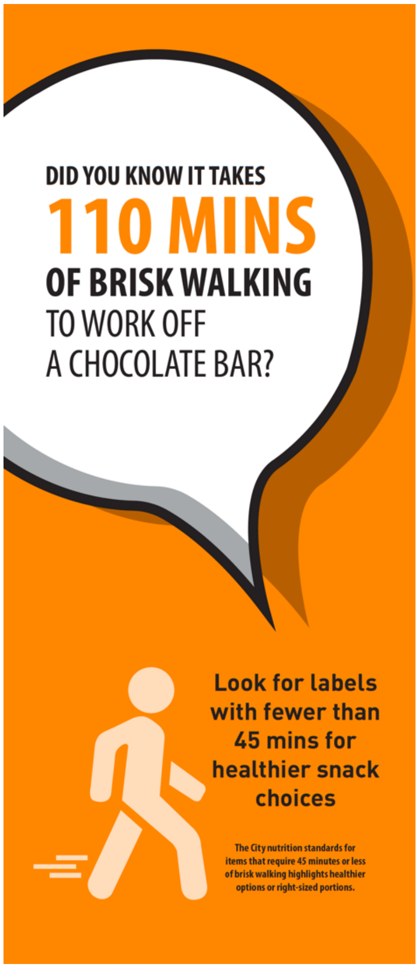

[3] Bleich SN, Herring BJ, Flagg DD, Gary-Webb TL. Reduction in purchases of sugar-sweetened beverages among low-income Black adolescents after exposure to caloric information. *Am J Public Health*. 2012;102(2):329-335.

© 2024 Gibson LA et al. *JAMA Network Open.*

A2. Example labels and posters in the field

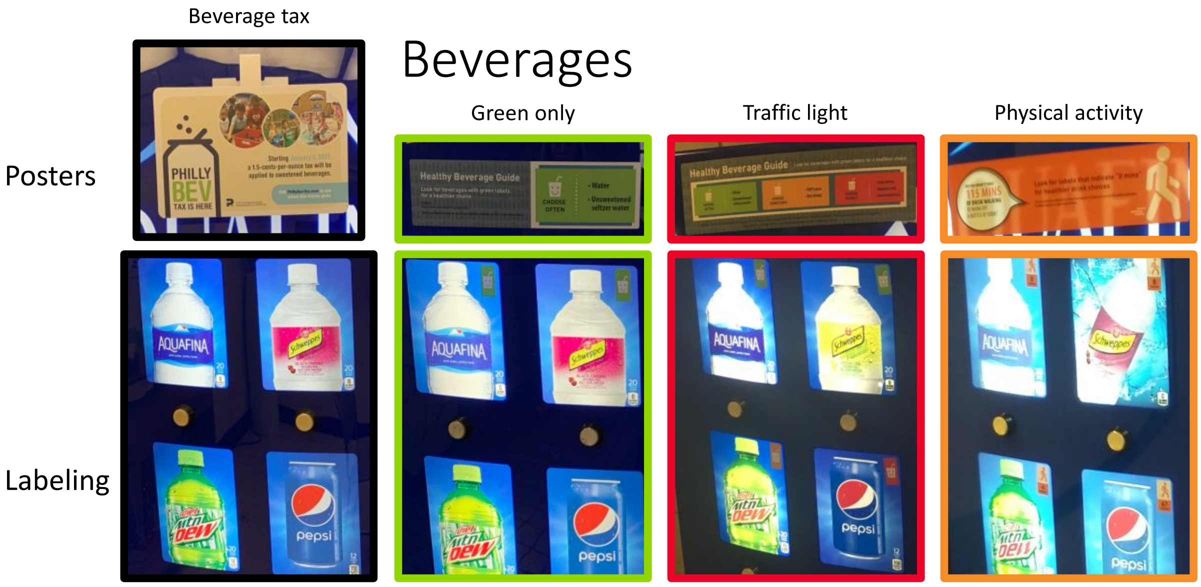

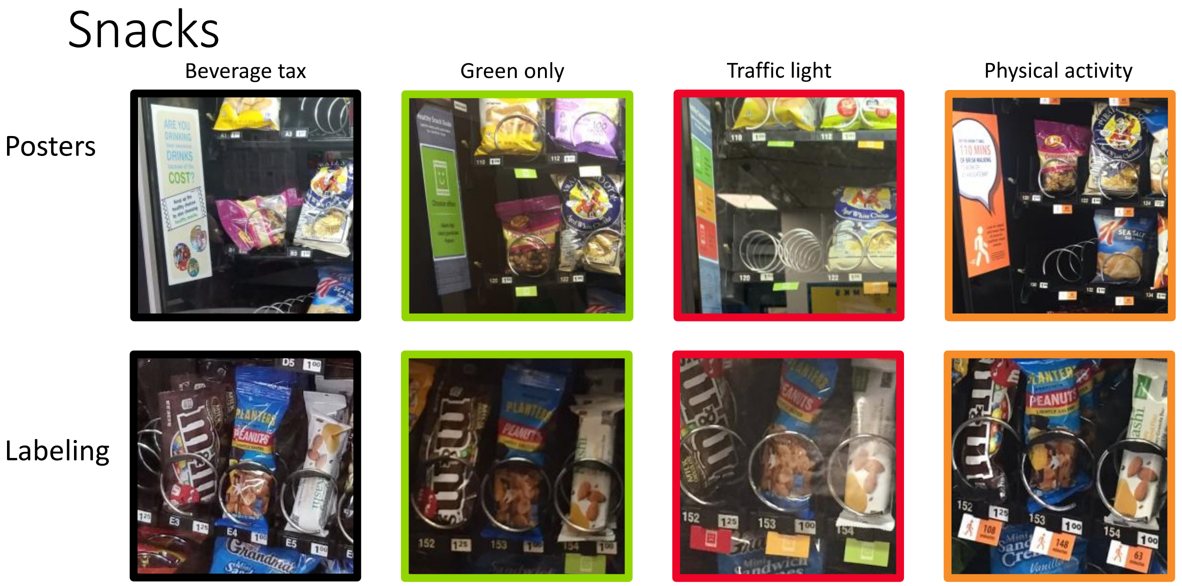

A3. Nutrition categorization

Item labels were created based on nutrition information gathered by research assistants from brand websites.

Beverage guidelines

For the green only and traffic light conditions, beverage nutrition classification was based on the guidelines in "Rethink Your Drink" from Building a Healthier Boston's Healthy Drink Toolkit. Beverages with 0-5 grams of sugar per 12 oz were labeled as most healthy or green (e.g., water,

© 2024 Gibson LA et al. *JAMA Network Open.*

seltzer). Beverages with 6-12 grams of sugar per 12 oz or artificial sweeteners were labeled as moderately healthy or yellow (e.g., diet soda, 100% fruit juice). Beverages with over 12 grams of sugar per 12 oz were labeled as unhealthy or red (e.g., sodas and juice drinks with added sugar). Therefore, every beverage labeled red and nearly[4] every beverage labeled yellow were subject to the Philadelphia sweetened beverage tax. Of the unique beverages available in each machine during the study, 28% were labeled green, 39% were labeled yellow, and 33% were labeled red.

Snack guidelines

Snack nutrition classification was based on calories, total fat, saturated fat, sugar, and whether or not ingredients included nuts, seeds, or fruit as in Franckle et al, 2018.[5] Snacks with fewer calories, less fat, and sugar were labeled as green, those with moderate levels were labeled as yellow, and those with the most calories, fat and sugar were labeled as red (see below). Nuts, seeds, and fruits without added sugar were allowed to have higher levels of fat and sugar, as long as the calories matched that category. All gums and mints were set to yellow even though they met the criteria for green because they have no nutritional value (no fiber, protein, or vitamins). Of the unique snacks available in each machine during the study, 47% were labeled green, 37% were labeled yellow, and 16% were labeled red.

Snack healthfulness guidelines (green, yellow, red)

	Green:	Yellow:	Red:
Calories	≤ 250	> 250 ≤ 350	> 350
Total Fat (g)	≤ 5g (except nuts/seeds)	> 5g ≤ 9g (except nuts/seeds)	> 9g
Sat Fat (g)	≤ 1 (except nuts/seeds)	> 1 ≤ 3 (not a nut/seed)	> 3 (not a nut/seed)
Sugar (g)	≤ 18 (except fruit, nuts and seeds with no added sugar)	> 18 (includes only fruit and nuts/seeds with added sugar)	> 18

A4. Physical activity equivalent calculations

In contrast to the two healthfulness conditions (green only and traffic light), the physical activity labels were based solely on product calories. Physical activity equivalent labels translated calories into the number of minutes of brisk walking required for a person weighing 150 pounds to "work off" those calories. Calculations started with assessing the increase in Basal Metabolic Rate (BMR) for brisk walking (3.2) over sitting or watching TV (1.2) = 2. These values are from the physical activity levels in the WHO/FAO report (Annex 5).

$$\text{Minutes} = \frac{\text{Calories} * 24\text{ hours} * 60\text{ minutes}}{(\text{BMR slope }(14.9375) * \text{Weight }(68\text{ kg}) + \text{Intercept }(589.40)) * \text{Walking increase }(2)}$$

[4] Only orange juice and an unsweetened flavored water (8% of yellow beverage sales) were not taxed.

[5] Franckle RL, Levy DE, Macias-Navarro L, Rimm EB, Thorndike AN. Traffic-light labels and financial incentives to reduce sugar-sweetened beverage purchases by low-income Latino families: a randomized controlled trial. *Public Health Nutrition.* 2018;21(8):1426-1434.

© 2024 Gibson LA et al. *JAMA Network Open.*

All gums and mints were set to 5 minutes of physical activity because we did not expect someone to eat the whole package at once. Of the unique beverages available in each machine during the study, the median physical activity equivalent was 0 minutes. Among those beverages with physical activity equivalents > 0 minutes, the mean was 67 minutes (SD=33, median = 67, range = 4-135). Of the unique snacks available in each machine during the study, the mean physical activity equivalent was 74 minutes (SD=43, median = 63, range = 5-235).

© 2024 Gibson LA et al. *JAMA Network Open.*

eAppendix 2. Sales Data

B1. Data excluded from vending machine analyses

Data were first pooled across intervention arms to conduct quality checks. We excluded an outlier month of sales from one beverage machine at baseline and another month during the intervention with more than 1.5 times the number of items sold relative to the next highest month.

Beverages boxplot: max = 801, next closest outlier is 385.

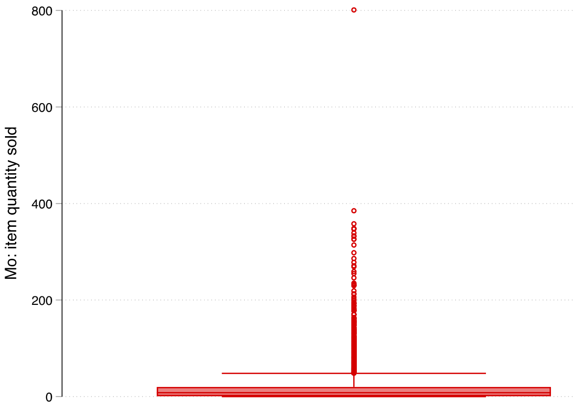

Snacks boxplot.

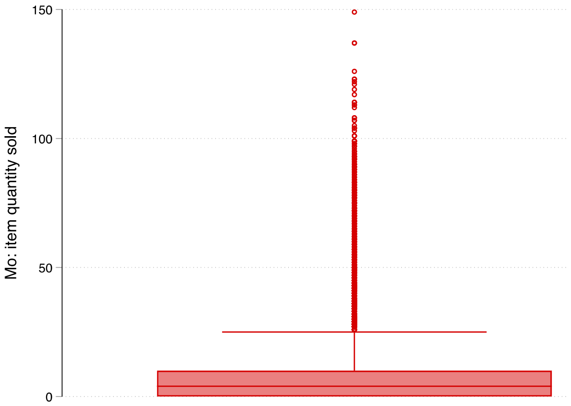

Beverage machines boxplot: max = 2093, next closest outlier is 1431.

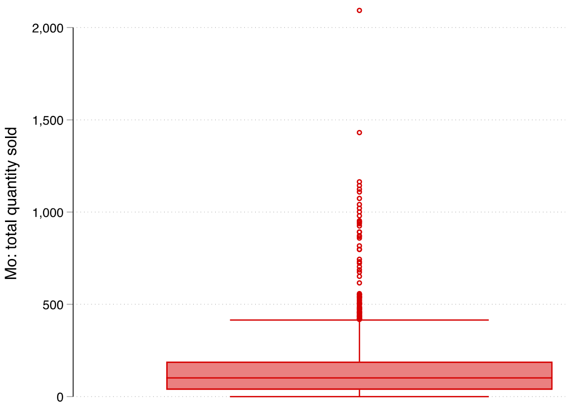

Snack machines boxplot.

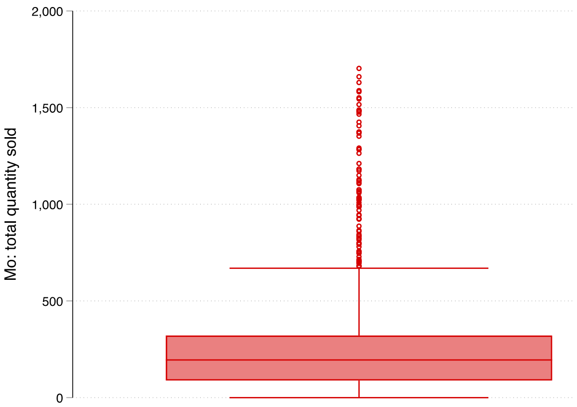

In addition, only 158 entries were dropped because they were listed as “unknown” (<1% of the 1.1M items sold), making it impossible to link them with nutrition information.

© 2024 Gibson LA et al. *JAMA Network Open.*

B2. Location balance tests

We expected different customer behavior at different types of locations and at machines with historically low versus high sales. People go to those locations for very different purposes and spend different amounts of time there. Therefore, we stratified by location type and baseline sales. The number of vending machine locations and the two stratification factors (location type and machine baseline sales) did not significantly differ across conditions in the analytic sample at baseline.

	Beverage (n=108 locations)				Snack (n=99 locations)			
	Beverage Tax N	Green only N	Traffic light N	Physical activity N	Beverage Tax N	Green only N	Traffic light N	Physical activity N
Total	27	32	22	27	22	29	22	26
% high sales	48%	56%	59%	48%	59%	62%	59%	50%
Types of locations								
Correctional facilities	2	4	2	3	2	3	2	3
Courts/offices	4	4	3	3	2	3	2	2
Large offices	6	5	4	5	5	5	4	6
Police/fire	6	6	5	7	5	6	5	7
Rec/Library/Other	9	13	8	9	8	12	9	8

Note. Chi-squared tests showed no significant differences by condition.

© 2024 Gibson LA et al. *JAMA Network Open.*

B3. Transaction-level overtime graphs

Beverages

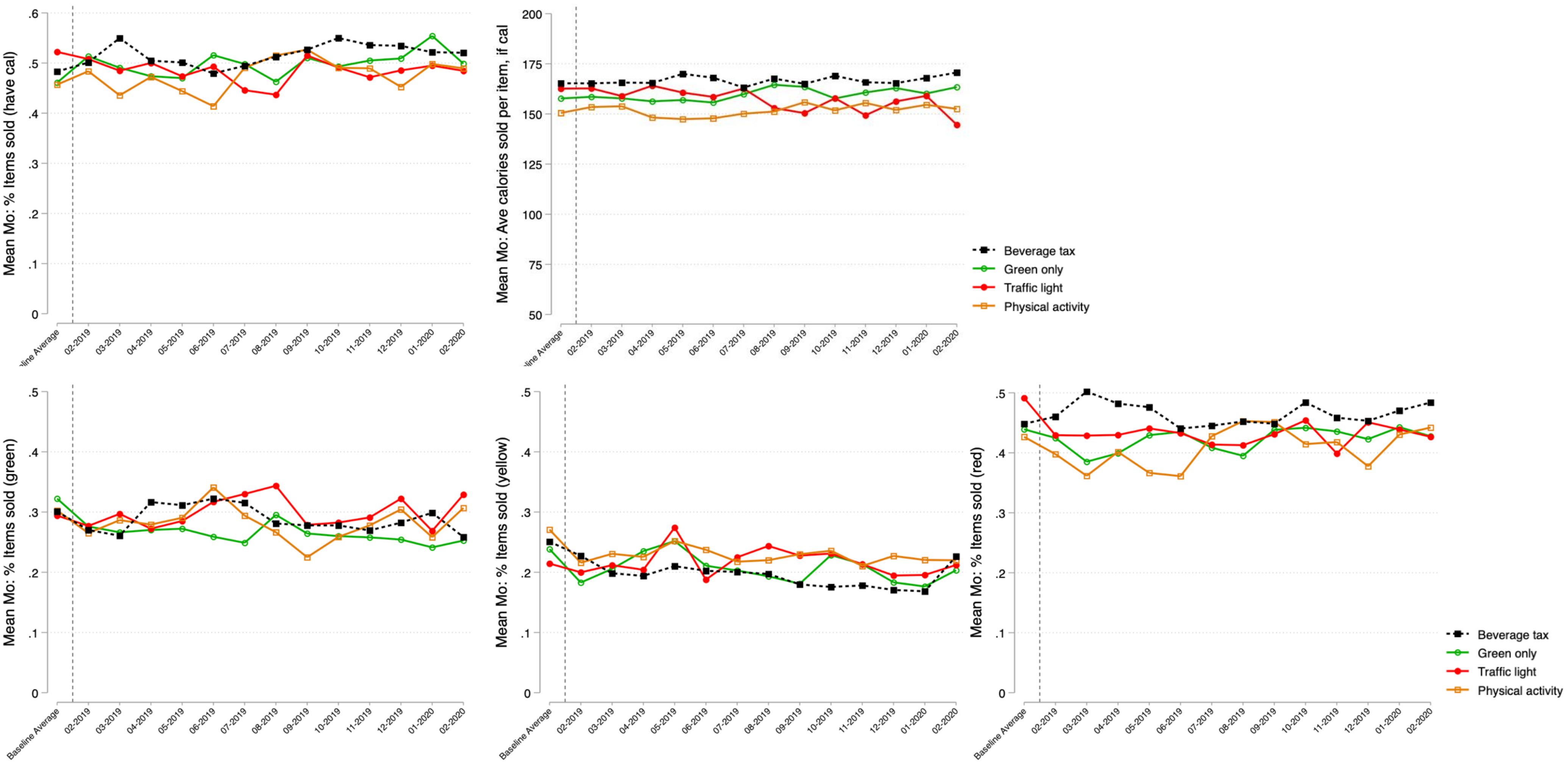

© 2024 Gibson LA et al. *JAMA Network Open.*

Snacks

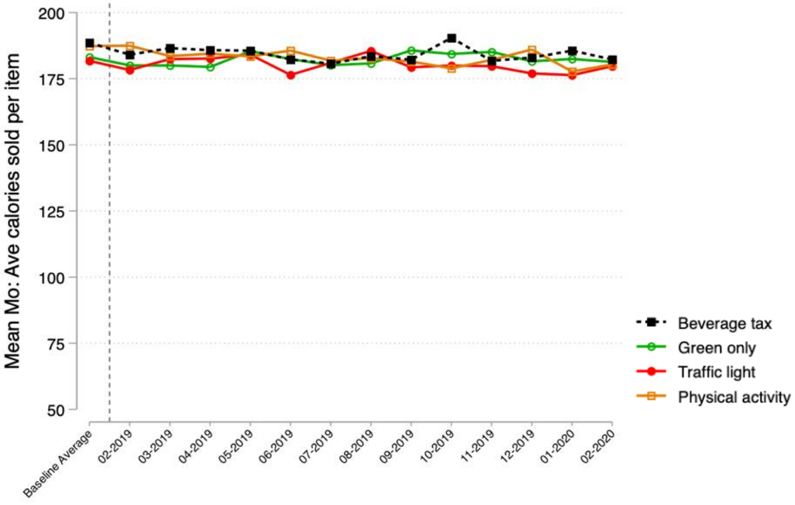

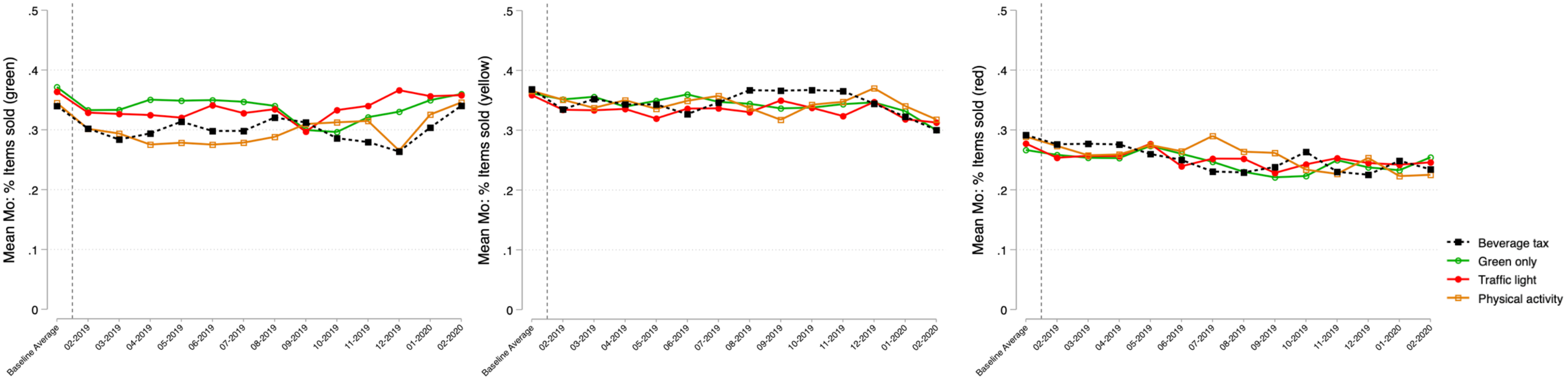

© 2024 Gibson LA et al. *JAMA Network Open.*

B4. Machine-level overtime graphs

Beverage machines

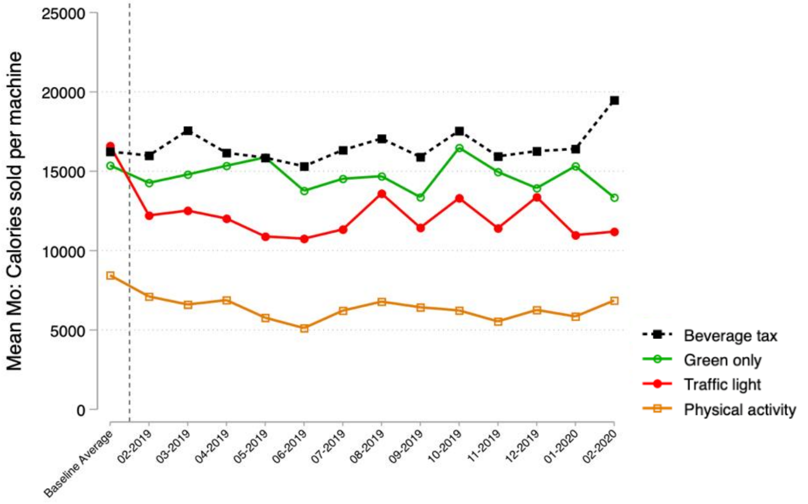

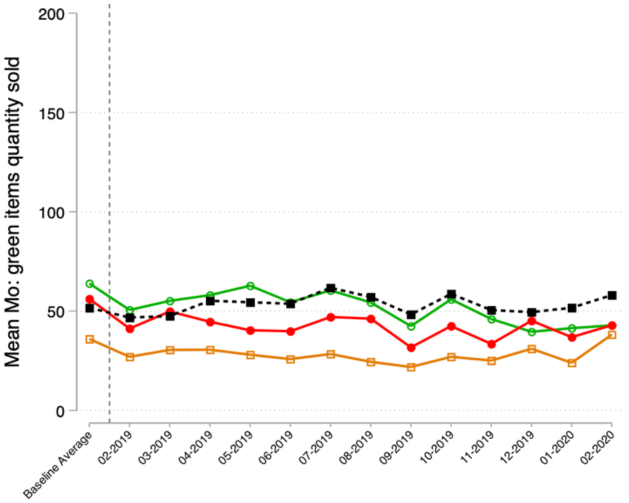

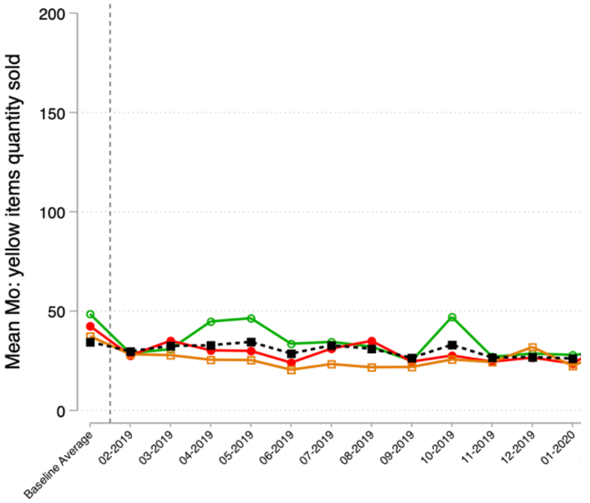

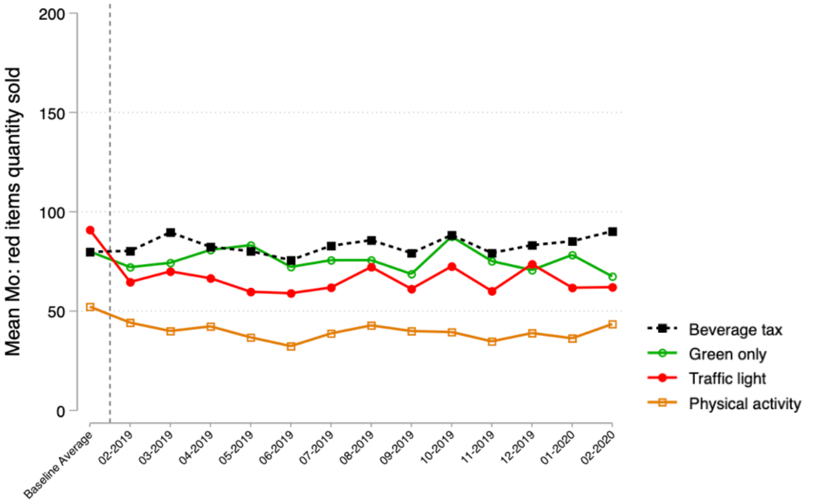

© 2024 Gibson LA et al. *JAMA Network Open.*

Snack machines

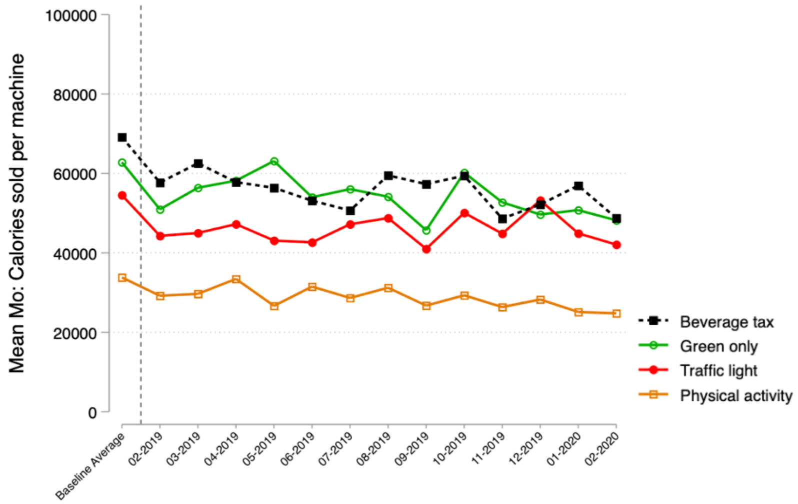

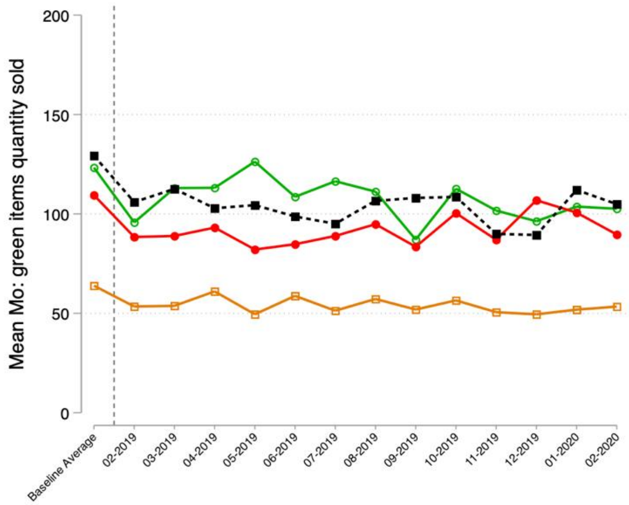

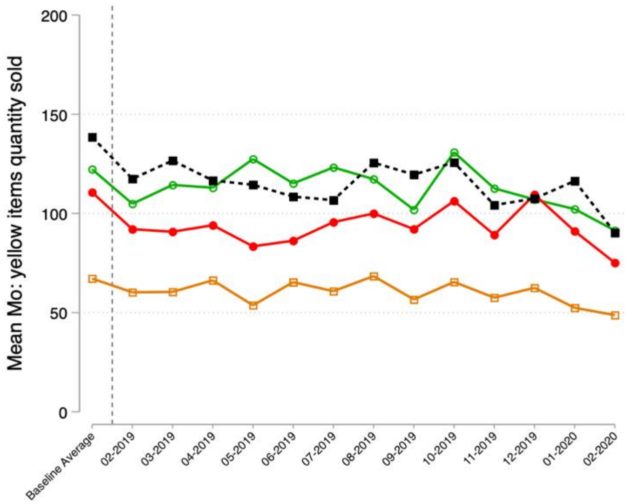

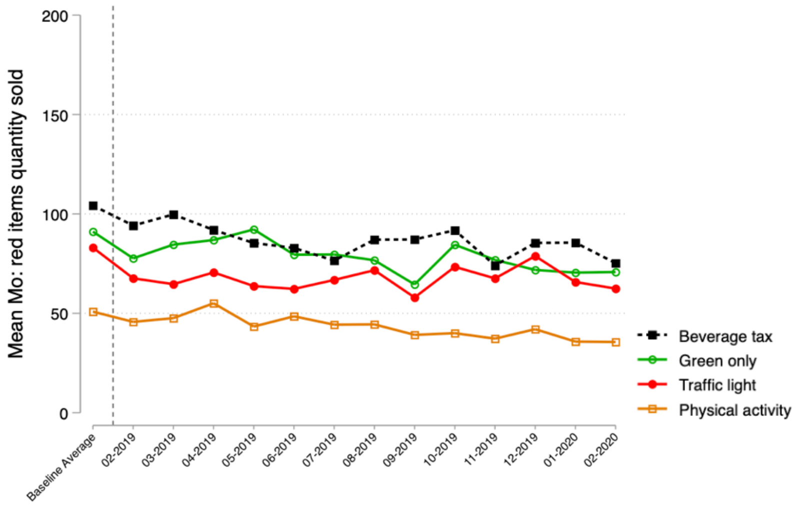

© 2024 Gibson LA et al. *JAMA Network Open.*

B5. Sensitivity 1: Dropping cumulative data

We conducted two sensitivity analyses. First, eleven beverage and five snack machines had large spikes in sales during a month that occurred after one or more months when the machine had zero sales. The company could not explain these data points and we were concerned that they were not actual spikes in monthly sales, but rather the cumulative sum of that month and the previous months posting zero sales. Therefore, our first sensitivity analysis dropped these outlier months and the preceding months with zero sales.

Transaction-level monthly sales outcomes, conditional on sales

Condition	Has calories OR [95% CI]	Pred. margins % have cal	Calories, if calories b [95% CI]	Pred. margins	G (*ref R*) RRR [95% CI]	Y (*ref R*) RRR [95% CI]	Pred. margins % G	% Y	% R
Beverages	$Chi^2(3) = 4.00$		$Chi^2(3) = 6.12$		$Chi^2(6) = 11.93$				
Beverage tax	*Ref*	52.3	*Ref*	178.8	*Ref*	*Ref*	32.7	18.4	48.8
Green only	0.98 [0.88, 1.09]	51.7	-1.35 [-5.82, 3.13]	177.4	0.94 [0.84, 1.06]	1.18 [1.02, 1.38]	30.4	21.4	48.2
Traffic light	0.90 [0.81, 1.01]	49.8	-4.89 [-10.32, 0.54]	173.9	1.01 [0.90, 1.13]	1.25 [1.05, 1.48]	31.5	21.8	46.7
Physical activity	0.89 [0.76, 1.06]	49.6	-3.96 [-8.39, 0.47]	174.8	1.04 [0.89, 1.20]	1.31 [1.02, 1.69]	31.7	22.4	45.8
Snacks			$Chi^2(3) = 3.08$		**$Chi^2(6) = 13.80$***				
Beverage tax	–	–	*Ref*	181.6	*Ref*	*Ref*	**34.5[b,c]**	37.5	**28.0**
Green only	–	–	0.68 [-1.81, 3.17]	182.3	**1.10 [1.03, 1.17]**	1.08 [1.00, 1.16]	**35.7[a]**	37.9	**26.4**
Traffic light	–	–	0.81 [-1.98, 3.59]	182.4	**1.09 [1.02, 1.15]**	1.03 [0.94, 1.12]	**36.0[a]**	37.0	**27.0**
Physical activity	–	–	-2.04 [-5.47, 1.38]	179.6	1.02 [0.92, 1.14]	1.05 [0.96, 1.15]	34.4	38.3	27.3

Note. These sensitivity models drop potentially cumulative data. Calories: *n* beverages=252,133 clustered on 106 locations, of which *n*=128,876 had calories; *n* snacks=368,844, clustered on 93 locations where there were sales. Overall predictive margins for calories from non-caloric and caloric beverages: Beverage tax=93.3, Green only=91.6, Traffic light=86.6, Physical activity=86.7. Product healthfulness, sales in locations where unhealthy (red) items were available: *n* beverages=245,597 clustered on 102 locations; *n* snacks=354,148 clustered on 89 locations. Covariates in all models included: time (categorical month) and several baseline machine-level characteristics: average corresponding outcome across previous 9 months, average number of unique red items available, beverages smaller than 20oz ever available (beverage analyses only), binary sales (high/low), 4 dummies for 5 location types, and the sales x location type interaction. **Bold** indicates significant differences. Super-scripts indicate either which conditions differ or which conditions differ in the contrast of %R(ed)=% unhealthy versus either %G(reen)=% healthy or %Y(ellow)=% moderately healthy, using Bonferroni-Holm adjustment: (Beverage tax=a, Green only=b, Traffic light=c, Physical activity=d); OR=Odds ratio; RRR=Relative risk ratio; Pred. margins = Predictive margins; *Ref*=Reference group; * $p<.05$, ** $p<.01$

© 2024 Gibson LA et al. *JAMA Network Open.*

Machine-level monthly sales outcomes

Condition	N sold Mean Ratio [95% CI]	N sold Pred. margins	Calories sold Mean Ratio [95% CI]	Calories sold Pred. margins	N Green sold Mean Ratio [95% CI]	N Green sold Pred. margins	N Yellow sold Mean Ratio [95% CI]	N Yellow sold Pred. margins	N Red sold Mean Ratio [95% CI]	N Red sold Pred. margins
Beverage machines	$Chi^2(3) = 6.76$		**$Chi^2(3) = 10.52$***		$Chi^2(3) = 2.84$		$Chi^2(3) = 1.72$		**$Chi^2(3) = 12.11$****	
Beverage tax	*Ref*	190.0	*Ref*	**20,466[d]**	*Ref*	57.4	*Ref*	54.2	*Ref*	**103.9[c,d]**
Green only	0.84 [0.68, 1.03]	159.6	0.83 [0.65, 1.05]	16,904	0.86 [0.69, 1.08]	49.6	1.12 [0.86, 1.47]	60.8	0.80 [0.63, 1.03]	83.6
Traffic light	0.89 [0.74, 1.08]	169.6	0.75 [0.59, 0.94]	15,293	0.91 [0.71, 1.18]	52.5	1.03 [0.82, 1.30]	55.9	**0.70 [0.55, 0.89]**	**72.3[a]**
Physical activity	0.79 [0.64, 0.98]	150.7	**0.68 [0.52, 0.89]**	**13,934[a]**	0.83 [0.65, 1.07]	47.9	0.90 [0.69, 1.18]	48.9	**0.69 [0.53, 0.90]**	**72.1[a]**
Snack machines	$Chi^2(3) = 0.88$		$Chi^2(3) = 2.84$		$Chi^2(3) = 4.74$		$Chi^2(3) = 2.12$		$Chi^2(3) = 4.31$	
Beverage tax	*Ref*	277.2	*Ref*	46,828	*Ref*	90.5	*Ref*	96.8	*Ref*	70.7
Green only	1.07 [0.83, 1.37]	296.0	1.17 [0.95, 1.44]	54,894	1.22 [1.00, 1.49]	110.1	1.17 [0.94, 1.45]	112.9	1.16 [0.91, 1.49]	82.2
Traffic light	1.10 [0.83, 1.46]	305.7	1.11 [0.85, 1.46]	52,175	1.19 [0.91, 1.55]	107.6	1.07 [0.81, 1.42]	103.8	1.28 [0.96, 1.70]	90.2
Physical activity	0.99 [0.74, 1.32]	273.1	1.02 [0.78, 1.35]	47,957	1.01 [0.75, 1.36]	91.6	1.05 [0.80, 1.38]	101.7	1.00 [0.73, 1.36]	70.3

Note. These sensitivity models drop potentially cumulative data. Total quantity sold: *n* beverage machines=1,795 clustered on 107 locations; *n* snack machines=1,460 clustered on 99 locations. Monthly machine-level calories and quantity colors sold: *n* beverage machines=1,759 clustered on 106 locations; *n* snack machines=1,395 clustered on 96 locations. Covariates in all models included: time (categorical month) and several baseline machine-level characteristics: average corresponding outcome across previous 9 months, average number of unique red items available, beverages smaller than 20oz ever available (beverage analyses only), binary sales (high/low), 4 dummies for 5 location types, and the sales x location type interaction. **Bold** indicates significant differences. Super-scripts indicate which conditions differ, using Bonferroni-Holm adjustment: (Beverage tax=a, Green only=b, Traffic light=c, Physical

© 2024 Gibson LA et al. *JAMA Network Open.*

activity=d); Green=healthy, Yellow=moderately healthy, Red=unhealthy; Pred. margins=Predictive margins; *Ref*=Reference group; **p*<.05, ***p*<.01

B6. Sensitivity 2: Dropping products and machines that did not get treatment

Our second sensitivity analysis dropped seven products that were not consistently labeled (gums and mints, <1% of all sales) and 10 machines that were mislabeled or where labels were absent.

Transaction-level monthly sales outcomes, conditional on sales

Condition	Has calories OR [95% CI]	Pred. margins % have cal	Calories, if calories b [95% CI]	Pred. margins	G (*ref R*) RRR [95% CI]	Y (*ref R*) RRR [95% CI]	Pred. margins % G	% Y	% R
Beverages	$Chi^2(3)$ = 4.81		$Chi^2(3)$ = 5.91		$Chi^2(6)$ = 11.52				
Beverage tax	*Ref*	52.2	*Ref*	177.8	*Ref*	*Ref*	31.9	19.2	48.9
Green only	0.98 [0.88, 1.10]	51.7	-2.99 [-5.88, -0.09]	174.8	0.95 [0.84, 1.07]	1.16 [1.00, 1.35]	29.9	21.9	48.2
Traffic light	0.90 [0.81, 1.00]	49.6	-2.47 [-5.51, 0.58]	175.3	1.02 [0.91, 1.14]	1.23 [1.05, 1.45]	30.9	22.4	46.7
Physical activity	0.89 [0.75, 1.05]	49.4	-2.83 [-6.15, 0.49]	174.9	1.05 [0.91, 1.21]	1.30 [1.02, 1.66]	31.2	23.1	45.7
Snacks			$Chi^2(3)$ = 4.87		**$Chi^2(6)$ = 18.36****				
Beverage tax	–	–	*Ref*	184.5	*Ref*	*Ref*	**35.0[b]**	36.5	**28.5**
Green only	–	–	0.49 [-1.54, 2.51]	185.0	**1.12 [1.05, 1.19]**	1.09 [1.01, 1.17]	**36.5[a]**	37.0	**26.6**
Traffic light	–	–	0.43 [-2.45, 3.30]	185.0	1.08 [1.02, 1.15]	1.02 [0.93, 1.12]	36.5	36.0	27.5
Physical activity	–	–	-2.94 [-6.26, 0.37]	181.6	1.02 [0.92, 1.14]	1.06 [0.97, 1.15]	34.8	37.6	27.7

Note. These sensitivity models drop products and machines that did not receive the treatment with fidelity. Calories: *n* beverages=247,844 clustered on 104 locations, of which *n*=126,388 had calories; *n* snacks=352,445, clustered on 90 locations where there were sales. Overall predictive margins for calories from non-caloric and caloric beverages: Beverage tax=92.5, Green only=90.2, Traffic light=86.8, Physical activity=86.3. Product healthfulness, sales in locations where unhealthy (red) items were available: *n* beverages=241,308 clustered on 100 locations; *n* snacks=338,162 clustered on 86 locations. Covariates in all models included: time (categorical month) and several baseline machine-level characteristics: average corresponding outcome across previous 9 months, average number of unique red items available, beverages smaller than 20oz ever available (beverage analyses only), binary sales (high/low), 4 dummies for 5 location types, and the sales x location type interaction. **Bold** indicates significant differences. Super-scripts indicate either which conditions differ or which conditions differ in the contrast of %R(ed)=% unhealthy versus either %G(reen)=% healthy or %Y(ellow)=% moderately healthy, using Bonferroni-Holm adjustment: (Beverage tax=a, Green only=b, Traffic light=c, Physical activity=d); Pred. margins = Predictive margins; *Ref*=Reference group; * *p*<.05, ** *p*<.01

© 2024 Gibson LA et al. *JAMA Network Open.*

Machine-level monthly sales outcomes

Condition	N sold Mean Ratio [95% CI]	N sold Pred. margins	Calories sold Mean Ratio [95% CI]	Calories sold Pred. margins	N Green sold Mean Ratio [95% CI]	N Green sold Pred. margins	N Yellow sold Mean Ratio [95% CI]	N Yellow sold Pred. margins	N Red sold Mean Ratio [95% CI]	N Red sold Pred. margins
Beverage machines	**$Chi^2(3)$ = 8.57***		**$Chi^2(3)$ = 16.58****		$Chi^2(3)$ = 3.53		$Chi^2(3)$ = 2.11		**$Chi^2(3)$ = 16.89****	
Beverage tax	*Ref*	**192.8[d]**	*Ref*	**23,265[d]**	*Ref*	54.9	*Ref*	38.9	*Ref*	**117.8[d]**
Green only	0.82 [0.67, 1.00]	157.4	0.72 [0.58, 0.90]	16,776	0.85 [0.67, 1.07]	46.5	1.03 [0.77, 1.39]	40.2	0.71 [0.55, 0.90]	83.3
Traffic light	0.89 [0.75, 1.06]	171.8	0.74 [0.60, 0.92]	17,327	0.94 [0.74, 1.20]	51.7	1.03 [0.81, 1.31]	40.3	0.70 [0.55, 0.88]	82.0
Physical activity	**0.79 [0.61, 0.95]**	**146.5[a]**	**0.63 [0.48, 0.82]**	**14,611[a]**	0.81 [0.62, 1.05]	44.4	0.83 [0.62, 1.11]	32.3	**0.64 [0.49, 0.83]**	**75.1[a]**
Snack machines	$Chi^2(3)$ = 0.49		$Chi^2(3)$ = 1.50		$Chi^2(3)$ = 2.88		$Chi^2(3)$ = 0.98		$Chi^2(3)$ = 3.42	
Beverage tax	*Ref*	279.1	*Ref*	47,673	*Ref*	92.1	*Ref*	94.3	*Ref*	72.4
Green only	1.00 [0.77, 1.30]	279.4	1.12 [0.90, 1.39]	53,354	1.17 [0.94, 1.45]	107.4	1.11 [0.88, 1.40]	104.8	1.09 [0.85, 1.41]	79.3
Traffic light	1.05 [0.79, 1.39]	293.1	1.07 [0.82, 1.41]	51,212	1.15 [0.87, 1.51]	105.8	1.03 [0.78, 1.37]	97.4	1.24 [0.93, 1.64]	89.5
Physical activity	0.95 [0.71, 1.28]	265.8	0.99 [0.75, 1.31]	47,354	0.99 [0.73, 1.33]	90.9	1.02 [0.77, 1.35]	96.4	0.96 [0.70, 1.31]	69.7

Note. These sensitivity models drop potentially cumulative data. Total quantity sold: *n* beverage machines=1,781 clustered on 105 locations; *n* snack machines=1,427 clustered on 96 locations. Monthly machine-level calories and quantity colors sold: *n* beverage machines=1,742 clustered on 104 locations; *n* snack machines=1,362 clustered on 93 locations. Covariates in all models included: time (categorical month) and several baseline machine-level characteristics: average corresponding outcome across previous 9 months, average number of unique red items available, beverages smaller than 20oz ever available (beverage analyses only), binary sales (high/low), 4 dummies for 5 location types, and the sales x location type interaction. **Bold** indicates significant differences. Super-scripts indicate which conditions differ, using Bonferroni-Holm adjustment: (Beverage tax=a, Green only=b, Traffic light=c,

© 2024 Gibson LA et al. *JAMA Network Open.*

Physical activity=d); Green=healthy, Yellow=moderately healthy, Red=unhealthy; Pred. margins = Predictive margins; *Ref*=Reference group; **p*<.05, ***p*<.01

© 2024 Gibson LA et al. *JAMA Network Open.*

B7. Secondary Analysis 1: 1-3 months vs 4-13 months

The first set of secondary analyses tested moderation of the intervention for all machine-level and transaction-level beverage and snack models by study time-period (first 3 months to the last 10 months).

Type	Model	df	Chi^2	*p*
Beverage transaction-level	Calories or not	3	2.11	.55
	Calories, if calories	3	**8.58***	**.04**
	Green or Yellow (vs Red) sold	6	10.39	.11
Snack transaction-level	Calories (all)	3	2.07	.56
	Green or Yellow (vs Red) sold	6	6.29	.39
Beverage machine-level	N sold	3	1.69	.64
	Calories (all)	3	2.04	.56
	Green sold	3	4.17	.24
	Yellow sold	3	0.92	.82
	Red sold	3	1.19	.76
Snack machine-level	N sold	3	4.11	.25
	Calories (all)	3	5.06	.17
	Green sold	3	2.71	.44
	Yellow sold	3	1.28	.73
	Red sold	3	7.57	.06

Note. Time (categorical month) was dropped from all models to accommodate this moderation. Covariates in all models included: several baseline machine-level characteristics: average corresponding outcome across previous 9 months, average number of unique red items available, beverages smaller than 20oz ever available (beverage analyses only), binary sales (high/low), 4 dummies for 5 location types, and the sales x location type interaction. **Bold** indicates significant differences. None of the 6 pairwise contrasts for "Calories, if calories" were significant. * $p<.05$, ** $p<.01$

© 2024 Gibson LA et al. *JAMA Network Open.*

B8. Secondary Analysis 2: Machine-level monthly dollar sales

The second set of secondary analyses tested machine-level sales in dollars for beverages and snacks.

Condition	Dollars Mean Ratio [95% CI]	Predictive margins	Sensitivity 1: Dollars Mean Ratio [95% CI]	Predictive margins	Sensitivity 2: Dollars Mean Ratio [95% CI]	Predictive margins
Beverage machines	$Chi^2(3) = 7.52$		$Chi^2(3) = 6.07$		**$Chi^2(3) = 8.25$***	
Beverage tax	*Ref*	$250.56	*Ref*	$256.44	*Ref*	**$255.13[d]**
Green only	0.93 [0.75, 1.14]	$232.12	0.95 [0.77, 1.16]	$242.48	0.88 [0.72, 1.09]	$225.32
Traffic light	0.86 [0.71, 1.04]	$215.17	0.86 [0.70, 1.05]	$219.45	0.87 [0.72, 1.06]	$222.67
Physical activity	0.75 [0.60, 0.95]	$189.11	0.79 [0.64, 0.98]	$202.23	**0.75 [0.60, 0.94]**	**$190.67[a]**
Snack machines	$Chi^2(3) = 2.93$		$Chi^2(3) = 2.82$		$Chi^2(3) = 1.39$	
Beverage tax	*Ref*	$265.45	*Ref*	$266.16	*Ref*	$267.84
Green only	1.18 [0.96, 1.45]	$312.78	1.18 [0.95, 1.44]	$312.86	1.12 [0.90, 1.39]	$300.12
Traffic light	1.12 [0.85, 1.46]	$296.35	1.12 [0.86, 1.47]	$298.01	1.08 [0.82, 1.42]	$289.12
Physical activity	1.04 [0.78, 1.37]	$274.86	1.03 [0.78, 1.37]	$275.21	1.00 [0.76, 1.33]	$269.10

Note. Monthly machine-level sales in dollars: *n* beverage machines=1,817 clustered on 107 locations; *n* snack machines=1,411 clustered on 96 locations. Sensitivity 1 drops products and machines that did not receive the treatment with fidelity: *n* beverage machines=1,759 clustered on 106 locations; *n* snack machines=1,395 clustered on 96 locations. Sensitivity 2 drops potentially cumulative data: *n* beverage machines=1,742 clustered on 104 locations; *n* snack machines=1,362 clustered on 93 locations. Covariates in all models included: time (categorical month) and several baseline machine-level characteristics: average corresponding outcome across previous 9 months, average number of unique red items available, binary sales (high/low), 4 dummies for 5 location types, and the sales x location type interaction. **Bold** indicates significant differences. Super-scripts indicate which conditions differ, using Bonferroni-Holm adjustment: (Beverage tax=a, Green only=b, Traffic light=c, Physical activity=d); *Ref*=Reference group; * $p<.05$, ** $p<.01$

© 2024 Gibson LA et al. *JAMA Network Open.*

eAppendix 3. Customer Purchase Assessments

C1. Purchase assessment procedures

The original sample size for purchase assessments was determined by the number of individuals required to detect an interaction of education level (2-levels) and labeling strategy (4-levels) on average calories per item purchased, separately for snack and beverage machines (2-levels). Assuming equal numbers of higher and lower educated individuals within labeling intervention, an effective sample size of 176 participants per high and low education groups within, for example, the Green only condition provide 80% power to detect a difference of 0.3 standard deviation units under the null hypothesis of a difference of 0 using a two-sided Student's t-test and two-sided alpha level of 0.05. A difference of 0.3 SD units amounts to 7-17 calories based on prior estimates of the standard deviation of calories per vending machine purchase.[6] To account for clustering of individuals by vending machine, we increased our target sample size by a factor of 1.05 to 185 based on an intraclass correlation of 0.01 and 6 purchasers per snack machine. That target would be reached by surveying 6 individuals each at 33 snack machines, resulting in 198 individuals per label condition and education status, and 1,584 individuals overall. For beverage machines we performed a similar calculation, resulting in 5 participants needed per machine across 38 machines per education status and label condition for a total of 1,520 participants across all levels of message conditions and education status to detect an interaction of the same magnitude. To account for potential missing data and incomplete surveys, we aimed to recruit just over 200 additional individuals for a total of 3,312 participants.

During data collection, our aim was to get 10 purchase assessments from every machine in the study to which we had access. During the entire study, 17 research assistants (RAs) participated in purchase assessments. Their data collection shifts were distributed to get assessments from all machines over the course of the intervention period. We tried to make sure each location was visited at least once a month, but prioritized locations without any purchase assessments over locations that already had many. RAs were posted near machines on weekdays, primarily in the morning, at lunch, and in the late afternoon. If no one was intercepted during those times at high yield machines, we tried alternate times of day (earlier in the morning, or later in the evening).

After adult customers completed their vending purchases, they were approached by study RAs who asked if they would be interested in completing a short survey about their purchase. If they were willing, the RA read the survey questions from their tablet and filled in the answers in Qualtrics. RAs were trained in group sessions where they practiced doing surveys with one another, and then were observed in the field during their first few shifts.

Customers could only participate in this study once. We had the full list of products available from the vendors. Therefore, the survey asked for the specific items they purchased. We used the nutrition facts from the brand website for each item to calculate calories and traffic light labeling for every product they purchased. After documenting their purchases, the survey asked about the intervention, whether it influenced their purchasing, and finally for their demographics. Participants who were not City employees were eligible for a small prize in exchange for completing the survey (city employees are not permitted to accept such prizes).

[6] Dingman DA, Schulz MR, Wyrick DL, Bibeau DL, Gupta SN. Does providing nutrition information at vending machines reduce calories per item sold? *J Public Health Policy*. 2015;36(1):110-122.

© 2024 Gibson LA et al. *JAMA Network Open.*

We began data collection on February 1, 2019 planning to collect surveys from 3,312 people over three years, but the study was cut short two years early in March 2020 by the Covid-19 pandemic. The 1,065 customer purchase assessments we did collect were from 105 beverage machines (Mean=4.6 per machine, Median=4, Min=1, Max=18) and 87 snack machines (Mean=7.3, Median=7, Min=1, Max=18). Of the 1,065 customers, 487 purchased a beverage (median=1, range=1-2) and 631 purchased a snack (median=1, range=1-7). Just 5% (n=53) purchased both on one trip.

We documented 604 refusals and 164 repeat customers. Thus, we approached people a total of 1,833 times and had a 67% response rate (33% refusals). Of those who responded, 13% were excluded because they were repeat customers.

C2. Covariate differences in purchase assessments vs sales analyses

For analyses of purchase assessments, first, there were only four location types because assessments were not done at court/office locations. Second, time was aggregated to quarters to reduce the number of parameters estimated with this smaller sample size. Third, because this analysis looks at beverage and snack outcomes combined, we adjusted for the sum of beverage and snack average calories sold and average total number of unique unhealthy items at baseline. And fourth, we did not adjust for whether small beverage sizes (< 20 oz) were ever available during baseline because that measure did not apply for snack-only purchase assessments or locations with snack-only machines.

C3. Calories per customer trip

Condition	Calories b [95% CI]	Pred. margins	Sensitivity 2: Calories b [95% CI]	Pred. margins	Sensitivity 3: Calories b [95% CI]	Pred. margins
	**$Chi^2(3)$ = 12.47****		**$Chi^2(3)$ = 12.42****		**$Chi^2(3)$ = 11.67****	
Beverage tax	*Ref*	179.9	*Ref*	181.7	*Ref*	174.3
Green only	-24.60 [-57.19, 7.99]	155.3	-24.90 [-57.45, 7.65]	156.8	-18.68 [-47.26, 9.90]	155.6
Traffic light	**-32.51 [-59.92, -5.11]**	**147.4[d]**	**-34.22 [-63.90, -6.89]**	**147.4[d]**	**-26.68 [-49.59, -3.77]**	**147.7[d]**
Physical activity	-2.05 [-30.75, 26.64]	**177.9[c]**	-4.36 [-33.00, 24.28]	**177.3[c]**	2.77 [-23.30, 28.83]	**177.1[c]**

Note. Outcome is sum of all beverage and snack calories sold per trip. *N*=1,010, clustered on 90 locations. The significant contrast between Traffic light and Physical Activity in the main analysis is -30.46 [-49.36, -11.56]. *Sensitivity 2* model drops products and machines that did not receive the treatment with fidelity: *n*=997, clustered on 88 locations. The significant contrast between Traffic light and Physical Activity in Sensitivity 2 is -29.85 [-48.81, -10.]. *Sensitivity 3* model drops one participant outlier who bought 7 fig bars on one trip: *n*=1009, clustered on 90 locations. The significant contrast between Traffic light and Physical Activity in Sensitivity 3 is -29.45 [-48.84, -10.05]. Purchase assessments were not permitted in the court/office locations and not conducted at correctional facilities with low traffic because a prison escort was required. Covariates included: time (categorical quarter), 5 baseline machine-level characteristics: average number of unique red items available, binary sales (high/low), 3 dummies for 4 location types, their interaction, and baseline levels of calories at the monthly level, and 3 binary participant characteristics: white, college or more education, and work for the city. **Bold** indicates significant differences. Super-scripts indicate which conditions differ, using Bonferroni-Holm

© 2024 Gibson LA et al. *JAMA Network Open.*

adjustment: (Beverage tax=a, Green only=b, Traffic light=c, Physical activity=d), Pred. margins = Predictive margins, *p<.05, **p<.01

C4. Moderation by education

An original aim of this study was to examine the degree to which education moderated our intervention effects, but the Covid-19 pandemic prevented us from reaching our target sample size. Because nutrition labeling systems can further health inequities if they are not easily understood by all groups,[7,8] we still explored education as a moderator. Moderation was tested by adding the interaction of education and condition and evaluating the joint significance of the interaction terms.

There was no significant moderation by education on effects of condition on total calories (interaction term: $Chi^2(3) = 0.55$, p=.91).

Condition	Calories b [95% CI]	Predictive margins
Lower education	$Chi^2(3) = 4.82$	
Beverage tax	*Ref*	200.5
Green only	-36.81 [-94.09, 20.46]	163.6
Traffic light	-44.22 [-100.45, 12.01]	156.2
Physical activity	-18.40 [-71.70, 34.89]	182.1
Higher education	**$Chi^2(3) = 10.25$***	
Beverage tax	*Ref*	167.6
Green only	-21.58 [-54.64, 11.47]	146.1
Traffic light	**-31.10 [-59.59, -2.62]**	**136.5^d**
Physical activity	9.73 [-19.28, 38.75]	**177.4^c**

Note. These subgroup analyses compare calories purchased among those with lower or higher amounts of education. Although there were differences in significance levels, the lack of significant moderation shows that estimates for lower and higher education are not significantly different from each other. Lower education: N=466, clustered on 87 locations. Higher education: N=544, clustered on 80 locations. Outcome is sum of all beverage and snack calories sold per trip. Covariates in all models included: time (categorical quarter), 5 baseline machine-level characteristics: average number of unique items available, binary sales (high/low), their interaction, 3 dummies for 4 location types, and baseline levels of outcomes at the monthly level, and 2 binary participant characteristics: white and work for the city. **Bold** indicates significant differences. Super-scripts indicate which conditions differ, using Bonferroni-Holm adjustment: (Beverage tax=a, Green only=b, Traffic light=c, Physical activity=d) *p<.05, **p<.01

[7] Persoskie A, Hennessy E, Nelson WL. US Consumers' Understanding of Nutrition Labels in 2013: The Importance of Health Literacy. *Prev Chronic Dis*. 2017;14:E86.
[8] Stran KA, Knol LL. Determinants of food label use differ by sex. *J Acad Nutr Diet*. 2013;113(5):673-679.

© 2024 Gibson LA et al. *JAMA Network Open.*

C5. Survey

Start of Block: Screener

Q1 Have you done this survey before?

- ◯ Yes (1)
- ◯ No (2)

End of Block: Screener

Start of Block: Snack items purchased

Display This Question:

If Have you done this survey before? = No

Q2 Number of snack items purchased:

▼ 0 (1) ... 7+ (8)

End of Block: Snack items purchased

Start of Block: Snack questions

Display This Question:

If Have you done this survey before? = No

Q3 Type of snack item:

- ◯ Baked chips (1)
- ◯ Granola/nut bars (2)
- ◯ Packaged nuts/trail mix (3)
- ◯ Gum/mints (4)
- ◯ Baked snacks (snapeas, chickpeas) (5)
- ◯ Chips (6)
- ◯ Candy/chocolate (7)
- ◯ Cookies (8)
- ◯ Other (9)

© 2024 Gibson LA et al. *JAMA Network Open.*

Display This Question:

If Loop current: Type of snack item: = Baked chips

And Have you done this survey before? = No

Q4 Name of baked chips snack item:

- ○ Baked Cheetos (2)
- ○ Baked Lay's (3)
- ○ Baked Lay's Sour Cream and Onion (52)
- ○ Baked Lay's BBQ (4)
- ○ Baked Ruffle (Plain) (5)

Display This Question:

If Loop current: Type of snack item: = Granola/nut bars

And Have you done this survey before? = No

Q5 Name of granola/nut bars snack item:

- ○ Fig Bar - Apple Cinnamon (12)
- ○ Fig Bar - Blueberry (13)
- ○ Kashi Honey Almond Flax Bar (18)
- ○ Kind Pressed Fruit Bar (52)
- ○ Nature Valley - Oats and Honey Granola Bar (20)
- ○ Nutri-Grain Bar (Strawberry) (21)
- ○ Special K Bar (22)

© 2024 Gibson LA et al. *JAMA Network Open.*

Display This Question:

If Loop current: Type of snack item: = Packaged nuts/trail mix

And Have you done this survey before? = No

Q6 Name of packaged nuts/trail mix snack item:

- ◯ Kars Cranberry Almond Delight (1)
- ◯ Kars Original Trail Mix (2)
- ◯ Planters Peanuts (3)
- ◯ Wonderful Pistachios (4)

Display This Question:

If Loop current: Type of snack item: = Baked snacks (snapeas, chickpeas)

Q7 Name of baked snacks (snapeas, chickpeas) snack item:

- ◯ GoBitos White Cheddar Chickpeas/Beanfield Bean & Rice chips (1)
- ◯ Harvest Snaps - Snappea Crisps (52)

Display This Question:

If Loop current: Type of snack item: = Chips

And Have you done this survey before? = No

Q8 Name of chips:

- ◯ Doritos - Cool Ranch (9)
- ◯ Doritos - Nacho Cheese (10)
- ◯ Fritos (14)
- ◯ Herr's Go Lite (53)
- ◯ Herr's Regular Chip (21)
- ◯ Herr's Pop BBQ (52)
- ◯ Herr's Pop Sea Salt (54)
- ◯ Lay's Kettle Cooked Jalapeno (26)
- ◯ Lay's Kettle Cooked Sea Salt (27)
- ◯ Real Deal - Veggie Chip (28)
- ◯ Sun Chips Garden Salsa (29)
- ◯ Sun Chips Harvest Cheddar (30)

© 2024 Gibson LA et al. *JAMA Network Open.*

Display This Question:

If Loop current: Type of snack item: = Candy/chocolate

And Have you done this survey before? = No

Q9 Name of candy/chocolate snack item:

- ○ M&M Peanut (1)
- ○ M&M Plain (2)
- ○ Peanut Chew (3)
- ○ Snickers (4)
- ○ Twix bar (52)

Display This Question:

If Loop current: Type of snack item: = Cookies

And Have you done this survey before? = No

Q10 Name of cookies:

- ○ Animal Crackers (1)
- ○ Grandma's Vanilla Cream Cookies (2)
- ○ Lance Peanut Butter Crackers (52)
- ○ Oreo 100 Calories (3)
- ○ Oreo Mini (4)
- ○ Rice Krispie Treat (5)
- ○ Snack Well (54)

© 2024 Gibson LA et al. *JAMA Network Open.*

Display This Question:

If Loop current: Type of snack item: = Other

And Have you done this survey before? = No

Q11 Name of other snack item:

- ○ Dole Fruit Squeeze (1)
- ○ Cheez-It (2)
- ○ Chex Mix - Honey Nut (7)
- ○ Pirate's Booty (21)
- ○ Popcorners Kettle (22)
- ○ Skinny Popcorn (57)
- ○ Snyder Olde Thyme (24)
- ○ Wheat Thins Popped (25)
- ○ Wise Popcorn Reduced Fat (26)
- ○ Oatmeal (52)
- ○ Dole Peach Fruit Cup (53)
- ○ Dole Mandarin Fruit Cup (54)
- ○ Tuna (55)

© 2024 Gibson LA et al. *JAMA Network Open.*

○ Other (27)

Display This Question:

If Loop current: Name of other snack item: = Other

And Have you done this survey before? = No

Q12 If other, what snack was purchased?

____

Display This Question:

If Have you done this survey before? = No

Q13 Size (only write number; participant can look at product to check)

____

Display This Question:

If Have you done this survey before? = No

Q14 Was the size in grams or ounces?

○ Grams (1)

○ Ounces (2)

Display This Question:

If Have you done this survey before? = No

Q15 Price of item (do not put $ sign):

____

Display This Question:

If Have you done this survey before? = No

Q16 How healthy do you think this item is (RA points to item participant purchased):

○ 1: Not at all healthy (1)

○ 2: Slightly unhealthy (2)

○ 3: Neither healthy nor unhealthy (3)

○ 4: Slightly healthy (4)

○ 5: Very healthy (5)

End of Block: Snack questions

Start of Block: Beverage items purchased

© 2024 Gibson LA et al. *JAMA Network Open.*

Display This Question:

If Have you done this survey before? = No

Q17 Number of beverage items purchased:

▼ 0 (1) ... 7+ (8)

End of Block: Beverage items purchased

Start of Block: Beverage questions

Display This Question:

If Have you done this survey before? = No

Q18 Type of beverage item:

- ◯ Soda (1)
- ◯ Diet Soda (2)
- ◯ Tea/Diet Tea (3)
- ◯ Water/Seltzer (4)
- ◯ Other (5)

Display This Question:

If Loop current: Type of beverage item: = Soda

Q19 Name of soda beverage item:

- ◯ Cherry Coke (6)
- ◯ Cherry Pepsi (7)
- ◯ Crush Orange Soda (8)
- ◯ Coke (9)
- ◯ Dr. Pepper (18)
- ◯ Fanta Grape Soda (19)
- ◯ Fanta Orange Soda (20)
- ◯ Fanta Pineapple Soda (21)
- ◯ Mountain Dew (29)
- ◯ Mug Root Beer (30)
- ◯ Pepsi (31)
- ◯ Seagrams Ginger Ale (34)
- ◯ Sprite (40)

© 2024 Gibson LA et al. *JAMA Network Open.*

Display This Question:

If Loop current: Type of beverage item: = Diet Soda

Q20 Name of diet soda beverage item:

- ◯ Coke Zero (10)
- ◯ Diet Cherry Pepsi (13)
- ◯ Diet Coke (14)
- ◯ Diet Dr. Pepper (15)
- ◯ Diet Mountain Dew (16)
- ◯ Diet Pepsi (17)
- ◯ Sprite Zero (37)

Display This Question:

If Loop current: Type of beverage item: = Tea/Diet Tea

Q21 Name of tea/diet tea beverage item:

- ◯ Brisk Tea (5)
- ◯ Fuse Iced Tea (22)
- ◯ Lipton Diet Green Tea (24)
- ◯ Lipton Green Tea (26)
- ◯ Lipton Peach Tea (27)
- ◯ Snapple Diet Peach (36)
- ◯ Snapple Lemon Tea (37)
- ◯ Snapple Mango Tea (38)
- ◯ Snapple Raspberry Tea (39)

© 2024 Gibson LA et al. *JAMA Network Open.*

Display This Question:

If Loop current: Type of beverage item: = Water/Seltzer

Q22 Name of water/seltzer beverage item:

- ◯ Aquafina Splash Raspberry (3)
- ◯ Aquafina Water (4)
- ◯ Dasani Sparkling Black Cherry (11)
- ◯ Dasani Water (12)
- ◯ Schweppes Lemon Lime (13)
- ◯ Schweppes Black Cherry (14)
- ◯ Seagrams Seltzer (15)

Display This Question:

If Loop current: Type of beverage item: = Other

Q23 Name of other beverage item:

- ◯ 100% Orange Juice (Minute Maid) (1)
- ◯ 100% Orange Juice (Tropicana) (2)
- ◯ G2 Grape (3)
- ◯ Lipton Fruit Punch (4)
- ◯ Minute Maid Fruit Punch (5)
- ◯ Vitamin Water Zero Lemon (6)
- ◯ Vitamin Water Zero Orange (7)
- ◯ Other (8)

Display This Question:

If Loop current: Name of other beverage item: = Other

Q24 If other, what beverage was purchased?

____

© 2024 Gibson LA et al. *JAMA Network Open.*

Display This Question:

If Have you done this survey before? = No

Q25 Size of beverage:

▼ 10 oz (1) ... 20 oz (4)

Display This Question:

If Have you done this survey before? = No

Q26 Price of item (do not include $ sign)

____

Display This Question:

If Have you done this survey before? = No

Q27 How healthy do you think this item is (RA should indicate item participant purchased) on a scale of 1 - 5?

- ◯ 1: Not at all healthy (1)
- ◯ 2: Slightly unhealthy (2)
- ◯ 3: Neither healthy nor unhealthy (6)
- ◯ 4: Slightly healthy (4)
- ◯ 5: Very healthy (7)

End of Block: Beverage questions

Start of Block: Vending machine visit frequency questions

Display This Question:

If Number of snack items purchased: != 0

And Have you done this survey before? = No

Q28 How often do you buy **snacks** from this vending machine?

- ◯ < 1 time per month or fewer (1)
- ◯ 2-3 times per month (2)
- ◯ 1-2 times per week (3)
- ◯ 3-6 times per week (4)
- ◯ 1 time per day (5)
- ◯ 2-3 times per day (6)
- ◯ 4+ times per day (7)
- ◯ Don't know (8)
- ◯ Refused (9)
- ◯ Not applicable (10)

© 2024 Gibson LA et al. *JAMA Network Open.*

Display This Question:

If Number of beverage items purchased: != 0

And Have you done this survey before? = No

Q29 How often do you buy **beverages** from this vending machine?

- < 1 time per month or fewer (1)
- 2-3 times per month (2)
- 1-2 times per week (3)
- 3-6 times per week (4)
- 1 time per day (5)
- 2-3 times per day (6)
- 4+ times per day (7)
- Don't know (8)
- Refused (9)
- Not applicable (10)

Display This Question:

If Have you done this survey before? = No

Q30 How often do you buy items from vending machines in general?

- < 1 time per month or fewer (1)
- 2-3 times per month (2)
- 1-2 times per week (3)
- 3-6 times per week (4)
- 1 time per day (5)
- 2-3 times per day (6)
- 4+ times per day (7)
- Don't know (8)
- Refused (9)

© 2024 Gibson LA et al. *JAMA Network Open.*

Display This Question:

If Have you done this survey before? = No

Q31 Which vending machine do you usually go to in this building?

- ◯ This one (1)
- ◯ Another one (2)
- ◯ Refused (3)

Skip To: End of Block If Which vending machine do you usually go to in this building? = This one

Skip To: End of Block If Which vending machine do you usually go to in this building? = Refused

---

Display This Question:

If Have you done this survey before? = No

Q32 The vending machine you typically go to is located on which floor in this building?

▼ Basement (1) ... 18th (20)

Display This Question:

If Have you done this survey before? = No

Q33 The vending machine you typically go to is located where on the floor in this building?

- ◯ Hallway (1)
- ◯ Gathering space with eating/food (e.g. staff dining room, café, breakroom with appliances, kitchen) (2)
- ◯ Gathering space without food (e.g. lobby, roll call, visitor’s lounge) (3)
- ◯ In a vending machine specific room/nook (nothing else in the vicinity) (5)
- ◯ Other (8)
- ◯ Refused (9)

Display This Question:

If The vending machine you typically go to is located where on the floor in this building? = Other

Q34 If other, how would you describe the location of the vending machines?

____

End of Block: Vending machine visit frequency questions

Start of Block: Questions about posters and labels

© 2024 Gibson LA et al. *JAMA Network Open.*

Display This Question:

If Have you done this survey before? = No

Q35 **RAs: For this next set of questions, participants should not be able to see posters/labels. These questions are meant to test their recall of the posters/labels.**

Display This Question:

If Have you done this survey before? = No

Q36 Did you notice any posters or labels displayed on the machines? By "posters" we mean large stickers on the machine, and by "labels" we mean small stickers next to products.

- ◯ Yes (1)
- ◯ No (2)
- ◯ Don't know (3)
- ◯ Refused (4)

Skip To: Q44 If Did you notice any posters or labels displayed on the machines? != Yes

Display This Question:

If Have you done this survey before? = No

Q37 Did any of the posters or labels convey information about the health or nutritional content of the food/beverage?

- ◯ Yes (1)
- ◯ No (2)
- ◯ Don't know (3)
- ◯ Refused (4)

Skip To: Q40 If Did any of the posters or labels convey information about the health or nutritional content of th... != Yes

Display This Question:

If Have you done this survey before? = No

Q38 Please describe what the posters said.
(RAs: Do not show options to participants. Participant answers

do not have to align precisely with choices, so long as general idea is the same)

- ◯ Healthy Snack Guide/Green labels (1)
- ◯ Healthy Snack Guide/Traffic lights (6)
- ◯ Physical activity markers (2)
- ◯ Beverage tax information (3)
- ◯ None of the above (4)
- ◯ Refused (7)

Display This Question:

If Have you done this survey before? = No

Q39 Please describe what the labels said.
(RAs: Do not show options to participants. Participant answers do not have to align precisely with choices, so long as general idea is the same.)

- ◯ Green labels (4)
- ◯ Traffic light colors/Emojis (7)
- ◯ Physical activity times (5)
- ◯ None of the above (6)
- ◯ Refused (8)

Display This Question:

If Have you done this survey before? = No

Q40 Did you notice any posters about the Philadelphia beverage tax?
(If participant is unfamiliar, you can explain it is a 1.5 cent per ounce tax on sugar-sweetened and artificially-sweetened beverages.)

- ◯ Yes (1)
- ◯ No (2)
- ◯ Don't know (3)
- ◯ Refused (4)

© 2024 Gibson LA et al. *JAMA Network Open.*

Display This Question:

If Have you done this survey before? = No

Q41 Did the posters or labels influence what you purchased?

- Yes (1)
- No (2)
- Did not see them (3)
- Don't know (4)
- Refused (5)

Display This Question:

If Did the posters or labels influence what you purchased? = Yes

Q42 To what extent did you use any of the health posters or labels on the machine to help you decide what to buy?

- 1: Not at all (1)
- 2: A little (2)
- 3: Somewhat (3)
- 4: A lot (7)
- Refused (8)

Display This Question:

If Did the posters or labels influence what you purchased? = Yes

Q43 Did these posters or labels make you more or less likely to buy items from the vending machines?

- 1: Much less likely (1)
- 2: Less likely (2)
- 3: Neither more nor less likely (3)
- 4: More likely (4)
- 5: Much more likely (5)
- Refused (6)

Page Break

Display This Question:

If Have you done this survey before? = No

Q44 **RAs: Now you can point out the labels and posters on the machine.**

© 2024 Gibson LA et al. *JAMA Network Open.*

Display This Question:

If Have you done this survey before? = No

Q45 How much did you trust the information on the posters and labels we have been talking about that are displayed on the vending machine?

- ◯ 1: Completely distrusted (1)
- ◯ 2: Somewhat distrusted (2)
- ◯ 3: Neither trusted nor distrusted (3)
- ◯ 4: Somewhat trusted (4)
- ◯ 5: Completely trusted (5)
- ◯ Refused (6)

Display This Question:

If Have you done this survey before? = No

Q46 Have you seen these posters/labels on other vending machines?

- ◯ Yes (1)
- ◯ No (2)
- ◯ Don't know (3)
- ◯ Refused (4)

Display This Question:

If Have you done this survey before? = No

Q47 Knowing that the City posted these posters and labels on the vending machine, to what extent do you think the City is trying to help improve your health?

- ◯ 1: Not at all (1)
- ◯ 2: A little (2)
- ◯ 3: Somewhat (3)
- ◯ 4: A lot (4)
- ◯ Refused (7)

© 2024 Gibson LA et al. *JAMA Network Open.*

Display This Question:

If Have you done this survey before? = No

Q48 Knowing that the City posted these posters and labels on the vending machine, to what extent do you find these messages annoying?

- ○ 1: Not at all annoying (1)
- ○ 2: A little annoying (2)
- ○ 3: Somewhat annoying (3)
- ○ 4: Very annoying (4)
- ○ Refused (6)

End of Block: Questions about posters and labels

Start of Block: Calorie labels

Display This Question:

If Have you done this survey before? = No

Q49 **RAs, read out loud:**
The next few questions will be about calorie labels. The vendors are required by law to display calorie labels next to products in the vending machines if it's not displayed on the front of the package.

Display This Question:

If Have you done this survey before? = No

Q50 Did you see any calorie labels on or near the vending machine products?

- ○ Yes (1)
- ○ No (3)
- ○ Refused (2)

Skip To: End of Block If Did you see any calorie labels on or near the vending machine products? = No

---

© 2024 Gibson LA et al. *JAMA Network Open.*

Display This Question:

If Have you done this survey before? = No

Q51 How likely are you to buy high-calorie items from the vending machines because of the calorie labels? (RAs: offer scale options)

- 1: Much less likely (1)
- 2: Less likely (2)
- 3: Neither more nor less likely (3)
- 4: More likely (4)
- 5: Much more likely (5)
- Refused (6)

Display This Question:

If Have you done this survey before? = No

Q52 How likely are you to buy low-calorie items from the vending machines because of the calorie labels? (RAs: offer scale options)

- 1: Much less likely (1)
- 2: Less likely (2)
- 3: Neither more nor less likely (3)
- 4: More likely (4)
- 5: Much more likely (5)
- Refused (6)

Page Break

End of Block: Calorie labels

Start of Block: Demographics

© 2024 Gibson LA et al. *JAMA Network Open.*

Display This Question:

If Have you done this survey before? = No

Q53 Age

- ◯ 18-24 years old (1)
- ◯ 25-34 years old (2)
- ◯ 35-44 years old (3)
- ◯ 45-54 years old (4)
- ◯ 55-64 years old (5)
- ◯ 65-74 years old (6)
- ◯ 75 years or older (7)
- ◯ Refused (8)

Display This Question:

If Have you done this survey before? = No

Q54 Sex

- ◯ Male (1)
- ◯ Female (2)
- ◯ Other (3)
- ◯ Don't know (4)
- ◯ Refused (5)

Display This Question:

If Have you done this survey before? = No

Q55 What is your race?

- ◯ Black (1)
- ◯ White (2)
- ◯ Asian (3)
- ◯ Other (4) ____
- ◯ Don't know (5)
- ◯ Refused (6)

© 2024 Gibson LA et al. *JAMA Network Open.*

Display This Question:

If Have you done this survey before? = No

Q56 Are you Hispanic?

- ◯ Yes (1)
- ◯ No (2)
- ◯ Don't know (3)
- ◯ Refused (4)

Display This Question:

If Have you done this survey before? = No

Q57 Highest level of education?

- ◯ Less than high school (1)
- ◯ High school or GED (2)
- ◯ Some college or Associate's degree (3)
- ◯ College or higher (4)
- ◯ Don't know (5)
- ◯ Refused (6)

Display This Question:

If Have you done this survey before? = No

Q58 What is your current marital status?

- ◯ Single (1)
- ◯ Married (2)
- ◯ Not married, living with partner (3)
- ◯ Refused (6)

© 2024 Gibson LA et al. *JAMA Network Open.*

Display This Question:

If Have you done this survey before? = No

Q59 What is the combined annual income of the adults (i.e., you and your partner if applicable) in your household before taxes?

- Less than $25,000 (1)
- $25,001-$50,000 (2)
- $50,001-$75,000 (3)
- $75,001-$100,000 (4)
- $100,001-$125,000 (5)
- $125,001-$150,000 (6)
- More than $150,000 (7)
- Refused (8)

Display This Question:

If Have you done this survey before? = No

Q60 Height in feet and inches?

	Feet	Inches
Height (1)	▼ 3 (1 ... Refused (6)	▼ 0 (1 ... Refused (13)

Display This Question:

If Have you done this survey before? = No

Q61 Weight in pounds?

- Pounds (1) ____
- Don't know (2)
- Refused (3)

Display This Question:

If Have you done this survey before? = No

Q62 Do you work for the City in any way (e.g., Civil servant, contractor, staff, elected official, etc.)?
RAs: if you are in a city employee only area, cannot give city employees item/prize.

- Yes (1)
- No (2)
- Don't know (3)
- Refused (4)

End of Block: Demographics

© 2024 Gibson LA et al. *JAMA Network Open.*

Start of Block: Thanks

Display This Question:

If Have you done this survey before? = No

Q63 **RAs, read out loud:**
Thanks for participating in our survey. If you talk to anyone about our survey, please don't mention it is about labeling of snacks and beverages because if they take the survey it would affect our data.

End of Block: Thanks

Start of Block: Notes section

Display This Question:

If Have you done this survey before? = No

Q64 Optional - notes about participant/intercept

____

____

____

____

____

End of Block: Notes section

Start of Block: Admin details

*

65 RA Name (first and last, capitalize first letters of first and last)

____

Q66 Time Block

- ○ First (8-10 AM) (5)
- ○ Morning (10:10 AM - 12:10 PM) (1)
- ○ Afternoon (12:20 PM - 2:20 PM) (2)
- ○ Evening (2:30 PM - 4:30 PM) (3)
- ○ Late (4:40 PM - 6:40 PM) (4)

Q67 Day of the week

- ○ Monday (1)
- ○ Tuesday (2)
- ○ Wednesday (3)
- ○ Thursday (4)
- ○ Friday (5)

© 2024 Gibson LA et al. *JAMA Network Open.*

68 Building

▼ ASD Modular Center (2) ... Other (76)

Display This Question:

If Building = Other

Q69 If other, what building are you in?

____

Q70 Floor

▼ Basement (1) ... 18th (20)

Q71 Location in Building

◯ Hallway (1)

◯ Gathering space with eating/food (e.g. staff dining room, café, breakroom with appliances, kitchen) (2)

◯ Gathering space without food (e.g. lobby, roll call, visitor's lounge) (3)

◯ In a vending machine specific room/nook (nothing else in the vicinity) (4)

◯ Other (8)

Display This Question:

If Location in Building = Other

Q72 If other, how would you describe the location of the vending machines?

____

End of Block: Admin details

Start of Block: Number of machines

Q73 Total number of machines used:

▼ 1 (2) ... 7+ (8)

End of Block: Number of machines

Start of Block: Machine ID

Q74 Machine ID

____

____

____

____

____

© 2024 Gibson LA et al. *JAMA Network Open.*

Q75 Type of machine

- ○ Beverage (1)
- ○ Snack (2)

Q76 Type of label/poster:

- ○ Green labels (2)
- ○ Traffic light (red, yellow, green) (1)
- ○ Physical activity (3)
- ○ Beverage tax (4)

End of Block: Machine ID

Start of Block: Reminder for RAs

Q77 **Reminder for RAs:**
Remember to check that you filled in all the information correctly (e.g. snack sizes, prices, etc).
If you used a filler for sizes, please be sure to go back and fix it.

End of Block: Reminder for RAs

© 2024 Gibson LA et al. *JAMA Network Open.*
