# Supplementary material for: Comparison of Sales From Vending Machines With 4 Different Food and Beverage Messages: A Randomized Trial
Source: JAMA Netw Open. 2024 May 8;7(5):e249438. doi: 10.1001/jamanetworkopen.2024.9438 (PMC11079689; doi:10.1001/jamanetworkopen.2024.9438)
Supplement: Supplement 3. — Data Sharing Statement [file jamanetwopen-e249438-s003.pdf]

Data Sharing Statement

Gibson. Comparison of Sales From Vending Machines With 4 Different Food and Beverage Messages. *JAMA Netw Open*. Published May 08, 2024.
doi:10.1001/jamanetworkopen.2024.9438

Data
Data available: No

Additional Information
Explanation for why data not available: The sales data are proprietary and therefore will not be shared. We will share the customer purchase assessment data.
